# Antennal Transcriptome Evaluation and Analysis for Odorant-Binding Proteins, Chemosensory Proteins, and Suitable Reference Genes in the Leaf Beetle Pest *Diorhabda rybakowi* Weise (Coleoptera: Chrysomelidae)

**DOI:** 10.3390/insects15040251

**Published:** 2024-04-07

**Authors:** Bo-Xin Xi, Xiao-Ning Cui, Su-Qin Shang, Guang-Wei Li, Youssef Dewer, Chang-Ning Li, Gui-Xin Hu, Yan Wang

**Affiliations:** 1 Biocontrol Engineering Laboratory of Crop Diseases and Pests of Gansu Province, College of Plant Protection, Gansu Agricultural University, Lanzhou 730070, China; sxqxbx@outlook.com (B.-X.X.); wangyl0211@163.com (Y.W.); 2 Key Laboratory for Grassland Ecosystem of Education Ministry, College of Pratacultural, Gansu Agricultural University, Lanzhou 730070, China; licn@gsau.edu.cn (C.-N.L.); huguixin@gsau.edu.cn (G.-X.H.); 3 College of Life Science, Yan’an University, Yan’an 716000, China; liguangwei@yau.edu.cn; 4 Phytotoxicity Research Department, Central Agricultural Pesticide Laboratory, Agricultural Research Center, 7 Nadi El-Seid Street, Giza 12618, Egypt; dewer72@yahoo.com

**Keywords:** *Diorhabda rybakowi*, antennal transcriptome, reference genes, odorant-binding proteins, chemosensory proteins, RT-qPCR analysis

## Abstract

**Simple Summary:**

The leaf beetle *Diorhabda rybakowi* Weise poses a severe threat to desert grasslands in Northwest China with its third instar larvae and adults, and even to the local ecological environment, attributed to its outbreak. Green-control attractants or repellents have become popular in recent years, but the olfactory mechanism of *D. rybakowi* is still unclear. Therefore, we preliminarily screened the best reference genes under different conditions and determined the bioinformatics characteristics and tissue expression profiles of *D. rybakowi* olfactory target genes. The recommended reference genes, *RPL13a* and *RPS18* for tissues and *RPL19* and *RPS18* for sexes, were determined. Notably, the transcriptional levels of *DrybOBP3*, *DrybOBP6*, *DrybOBP7*, *DrybOBP10*, *DrybOBP11*, *DrybCSP2,* and *DrybCSP5* among eleven odorant-binding proteins (OBPs) and six chemosensory proteins (CSPs) were significantly higher in the antenna. In summary, our study provides a strong basis for deepening the research of olfaction molecular mechanisms in *D. rybakowi*.

**Abstract:**

*Diorhabda rybakowi* Weise is one of the dominant pests feeding on *Nitraria* spp., a pioneer plant used for windbreaking and sand fixation purposes, and poses a threat to local livestock and ecosystems. To clarify the key olfactory genes of *D. rybakowi* and provide a theoretical basis for attractant and repellent development, the optimal reference genes under two different conditions (tissue and sex) were identified, and the bioinformatics and characterization of the tissue expression profiles of two categories of soluble olfactory proteins (OBPs and CSPs) were investigated. The results showed that the best reference genes were *RPL13a* and *RPS18* for comparison among tissues, and *RPL19* and *RPS18* for comparison between sexes. Strong expressions of *DrybOBP3*, *DrybOBP6*, *DrybOBP7*, *DrybOBP10*, *DrybOBP11*, *DrybCSP2*, and *DrybCSP5* were found in antennae, the most important olfactory organ for *D. rybakowi*. These findings not only provide a basis for further in-depth research on the olfactory molecular mechanisms of host-specialized pests but also provide a theoretical basis for the future development of new chemical attractants or repellents using volatiles to control *D. rybakowi*.

## 1. Introduction

In northwestern China, the desert grassland vegetation is scarce, and usually consists of few species, leading to a fragile ecological environment. *Nitraria* spp. includes *N*. *tangutorum*, *N*. *sphaerocarpa*, and *N. roborowskii*, which are the dominant genera in the local area. *Nitraria* spp. are the main windbreaking and sand-fixing plant due to their large root system, and also the main source of local livestock’ feed [1,2]. The leaf beetle *Diorhabda rybakowi* Weise (Coleoptera: Pteropodidae) is an oligophagous pest with *N*. *tangutorum* and *N*. *sphaerocarpa* as its main hosts. It is mainly distributed in Gansu, Ninxia, and Inner Mongolia [1,2]. The larvae and adults mainly feed on shoots and fresh leaves, resulting in leaf abscission and breakage, seriously jeopardizing the balance of local ecological environments [3]. Despite quite some research on the biology of this species, the population outbreak mechanism of *D. rybakowi* remains to be researched [3,4]. As such, clarifying the mechanism of chemoreception of *D. rybakowi* is required.

The chemical environment plays a critical role in the olfaction of insect communication, with host plants, food, habitat, and predators having their characteristic chemical signaling sources, thus creating a communication network (insect odorscapes) between insects and environments, e.g., volatile organic compounds (VOCs), volatile plant compounds (VPCs), and sex pheromones [5,6,7]. Various external chemicals can enter lymph through surface pores of hair-like or cone-like sensilla [8]. Soluble proteins, odorant-binding proteins (OBPs), and chemosensory proteins (CSPs) selectively bind and transport odorant molecules to the surface of olfactory neurons in the membrane, and odorant receptors convert captured chemicals into nerve impulses transmitted to the brain for regulation of various insect behavior [9,10]. OBPs and CSPs are the most important olfactory proteins, and only about 20 Coleoptera species have been identified, such as *Harmonia axyridis* [11], *Colaphellus bowringi* [12], *Callosobruchus maculatus* [13], *Anthonomus eugenii* [14], *Ophraella communa* [15], and *Galeruca daurica* [16]. OBPs generally have six highly conserved cysteines and three disulfide bridges. They are divided into four groups: classic OBPs, minus-C OBPs, plus-C OBPs, and atypical OBPs [17,18]. Based on previous studies, OBPs are involved in the chemosensory process of various behaviors, such as host recognition, pheromones, and oviposition site selection [19,20,21]. CSPs have four highly conserved cysteines. In *Athetis lepigone*, *AlepCSP2* had a strong binding affinity to two sex pheromones and five maize volatiles, suggesting that AlepCSP2 could play an important role in mating behaviors and host plant recognition [22]. OcomCSP12 of *O. communa* is specifically expressed in female ovaries, and silencing *OcomCSP12* significantly reduces ovulation in female ovaries, implying that that OcomCSP12 plays a critical role in the reproduction process [23]. In *Rhopalosiphum padi*, the overexpression of *RpadCSP7*, *RpadCSP4*, and *RpadCSP5* led to the increased insecticide resistance of this pest, indicating that CSPs can be highly bound to insecticides, thus reducing insecticide toxicity and increasing resistance to insecticides [24,25].

In assessing olfactory gene expressions via RT-qPCR, assay data accuracy is often affected by methodological errors, including cDNA synthesis efficiency, variations in RNA quality, and amplification efficiency [26]. Errors will result in unreliable target gene quantification. Consequently, RT-qPCR data require normalization, and the most effective way to normalize the data is by using a suitable reference gene [27,28]. In RT-qPCR studies, the common reference genes include *Actin* (*ACT*), *beta1-tubulin* (*TUB*), *ribosomal protein S18* (*RPS18*), *ribosomal protein L19* (*RPL19*), *ribosomal protein L13a* (*RPL13a*), *Syntaxin-6* (*SYN6*), and *glyceraldehyde-3-phosphate dehydrogenase* (*GAPDH*) [26,29,30,31]. However, most research has shown that reference genes always demonstrate different degrees of stability under biotic or abiotic stresses and are not similar among species [27]. Thus, selecting appropriate reference genes based on specific experimental conditions is essential [32,33].

Little is currently known regarding the major olfactory genes in *D. rybakowi*, and studies on reference gene stability have not yet been reported. In this study, we aim to identify suitable reference genes for quantification of gene expression in *D. rybakowi* and clarify the expression characteristics of OBP and CSP genes in different tissues of adult *D. rybakowi* with real time-qPCR. The findings will provide guidance not only for discovering the olfaction mechanism in this pest but also for developing green chemical repellents to control them.

## 2. Materials and Methods

### 2.1. Insect Rearing

Male and female *D. rybakowi* adults were collected from Minqin County, Gansu province, in July 2022. The adults were fed with fresh leaves of *N. tangutorum* in plastic tanks (diameter 10 cm, height 5 cm) and then placed in an artificial climate chamber (Shanhai Yuejin, model HQH-H500, Shanghai, China) with a temperature of 26 °C ± 1 °C, relative humidity of 65% ± 5%, and a light (L): dark (D) photoperiod of 16 h/8 h. All adults used in the experiment were 4-day-old virgin adults.

### 2.2. Sample Collection

For transcriptome sequencing, we dissected adult males and females under a dissecting microscope and collected 120 antennae each in 1.5 mL centrifuge tubes immersed in liquid nitrogen, stored at −80 °C, with three biological replicates for each male and female. To evaluate the suitable reference genes, we set up two experimental groups: tissue and sex. For tissue samples, the heads (90), thoraxes (50), abdomens (10), legs (100), and wings (80) were collected from male and female adults, respectively. For comparison between sexes, five male and five female adults were collected as a single biological replicate. Male and female adults were used to collect antennae (150), heads (90), thoraxes (50), abdomens (10), legs (100), and wings (80) for RT-qPCR. All samples mentioned above were quickly placed into 1.5 mL centrifuge tubes, immersed in liquid nitrogen, and stored at −80 °C until the RNA was extracted. All of the samples used in the experiment had three biological replications.

### 2.3. RNA Extraction and cDNA Synthesis

The total RNA was extracted using the RNAsimple Total RNA Kit (TIANGEN, Beijing, China) according to the manufacturer’s recommendation and dissolved in 40–100 μL RNase-free water, respectively. The RNA concentration was quantified using a Nanodrop 2000 spectrophotometer (Thermo Scientific, Waltham, MA, USA), and RNA integrity was analyzed with electrophoresis on 1.5% agarose gels. All samples of A_260_/A_280_ ratios from 1.8 to 2.2 with clear bands were retained. The male and female antenna samples (1.5 g per sample) were sent to Beijing Allwegene Technology Co., Ltd. (Beijing, China). for transcription determination. The reverse transcription of total RNA (500 ng) to cDNA using FastKing gDNA Eliminated-RT SuperMix (TIANGEN, Beijing, China) according to manufacturer’s instructions.

### 2.4. cDNA Library Construction, Assembly, and Gene Annotation

The transcriptome sequencing was divided into two groups of male and female antennae, with three biological replications in each group. The assays were performed using the Illumina Novaseq 6000 platform (Illumina, San Diego, CA, USA), and raw reads were obtained. Quality control was assessed using the Agilent Bioanalyzer 2100 system (Agilent Technologies, Santa Clara, CA, USA). The error rate should be less than 1.0%, and the values of Q20 and Q30 should be greater than 92%, which indicates good quality control of the sample data (Table S1). After removing adaptor sequences and low-quality sequences from the original reads, the Trinity v2.14.0 software [34] was used for assembling, CD-hit [35] was used for transcript categorization, the Corset program was used to remove redundancy, and the BUSCO v5.7.0 and RSEM software [36,37] were used to evaluate the quality of splicing and to calculate gene expression, respectively. The unigenes were annotated in seven commonly used databases, including the NCBI non-redundant protein sequences database (NR), the NCBI nucleotide sequences database (NT), the Eukaryotic Ortholog Groups/Clusters of Orthologous Groups of proteins database (KOG/COG) (http://www.ncbi.nlm.nih.gov/COG/, accessed on 22 January 2023), the Swiss-Prot database (http://www.ebi.ac.uk/uniprot/, accessed on 24 January 2023), the protein family database (Pfam) (http://pfam.sanger.ac.uk/, accessed on 25 January 2023), the Kyoto Encyclopedia of Genes and Genomes database (KEGG) (http://www.genome.jp/kegg/, accessed on 25 January 2023), and the Gene Ontology database (GO) (http://www.geneontology.org/, accessed on 25 January 2023). The annotated unigenes involved in GO, KEGG, and KOG/COG were classified to evaluate the function of the assembled genes.

### 2.5. Identification of Candidate Reference Genes and Olfactory Genes

According to the sequencing results of the non-reference transcriptome, olfactory genes were found with keyword searches in the annotation_merged.xls file, where the annotated functions of genes were retrieved from different databases. The selected candidate genes were rearranged in a new file. The LogView 2.3.1 software was used to open the unigene fasta file, and corresponding nucleic acid sequences of target genes with the Gene_IDs, which were screened out and sorted into a Word file [38]. The sequences of candidate reference genes and olfactory genes (OBPs and CSPs) were compared with Blastn and Blastx in NCBI. E-values, identity values, and insect species were checked to further verify the transcriptome screening data accuracy.

### 2.6. Sequence Analysis and Phylogenetic Tree Construction

Olfactory gene sequence analysis using the NCBI ORFfinder function (https://www.ncbi.nlm.nih.gov/orffinder/, accessed on 12 March 2023) was carried out to identify the candidate gene open reading frame. SignalP-4.1 (https://services.healthtech.dtu.dk/service.php?SignalP-4.1, accessed on 12 March 2023) used with the default arguments predicted the numbers and locations of candidate gene sequence signal peptides. Proteins were translated using Expasy (https://web.expasy.org/translate/, accessed on 14 March 2023). DNAMAN v9.0 software was used to compare gene nucleotide sequences. Phylogenetic trees were constructed based on high homology amino acid sequences in NCBI according to the Blastx results of the candidate gene nucleic acid sequences. Multiple comparisons of amino acid sequences were observed using MAFFT version 7.037 software [39]. Phylogenetic trees were constructed using the BioNJ algorithm in Seaview version 4.0, with confidence determined via the Bootstrap test repeated 1000 times [40,41]. FigTree version 1.43 was used to modify the phylogenetic tree, such as adjustments to mark color, branch type, and branch size. Finally, Photoshop CS6 was used to mark the pictures in detail.

### 2.7. Reverse-Transcription Quantitative PCR (RT-qPCR)

The primers for all genes involved in the experiment were designed for RT-qPCR using Primer3 (https://bioinfo.ut.ee/primer3-0.4.0/, accessed 25 March 2023) [42]. The primer parameters were as follows: an annealing temperature of 59 °C, a GC content of 40–50%, and a length of 17–25 bp. The amplified product sizes were 100–210 bp. All primers in the experiment were synthesized by Beijing Tsingke Biotech Co., Ltd. (Beijing, China).

The RT-qPCR experiments were performed on a LightCycler^®^ 96 Instrument (Roche, Basel, Switzerland) with 2×SYBR Green qPCR Master Mix (Servicebio, Wuhan, China) and optical 96-well plate. Each reagent required for the RT-qPCR assay was pre-mixed in advance. The assay mixture contained 10 μL of 2×SYBR Green qPCR Master Mix (None ROX), 1.0 μL of cDNA template, 0.4 μL of each primer pair, and 8.2 μL of RNase-free water in a total of 20 μL. The template for each gene was original cDNA (1000 ng/mL) diluted fivefold using a series of gradients (1:5, 1:25, 1:125, 1:625, and 1:3125) for establishments of standard melting curves. The amplification efficiency (E) and correlation coefficient (R^2^) were the most significant parameters evaluated, where the R^2^ was the slope of the amplification curve, and E was calculated as E = (10[−1/slope] − 1) × 100% [43]. The amplification used a two-step method, and the conditions were as follows: pre-degeneration, one cycle of 95 °C for 30 s; denaturation and annealing elongation, forty cycles of 95 °C for 15 s and 60 °C for 30 s; and melting curve, the temperature ranged from 55 °C to 95 °C, increasing by 0.5 °C per cycle. No template controls were included, and three biological replications and two technical replications were performed.

### 2.8. Analysis of the Stability of Candidate Reference Genes

Five programs, Delta Ct [44], GeNorm [45], NormFinder [46], BestKeeper [47], and RefFinder (https://blooge.cn/RefFinder/, accessed 20 July 2023) [48], were used evaluate reference gene stability. For GeNorm and NormFinder, original data were normalized using the formula Q = 2^−ΔCt^, in which ΔCt = each Ct-minCt. Q values were imported into macro GeNorm software developed based on EXCEL 2010, and the expression stability value (M) and pairwise comparison value (V_n_/V_n+1_) were obtained. An M value greater than 1.5 indicated that it was unsuitable as a candidate reference gene, and (V_n_/V_n+1_) with a critical value of 0.15 was used to determine the optimal number of reference genes. NormFinder calculated the differences between groups to order the candidate reference genes. Delta Ct, BestKeeper, and RefFinder analyzed the results directly from the raw data. Delta Ct used standard deviation to determine the candidate gene stability. In BestKeeper, the coefficients of variance (CV) and standard deviations (SD) were obtained, with the lowest value representing the highest stability. RefFinder integrated the results of the other four analysis methods for a comprehensive ranking of the candidate reference gene.

### 2.9. Tissue Expression Profiles of OBPs and CSPs

The 2^−ΔΔCt^ method was used to calculate the expression levels of OBPs and CSPs in different adult tissues [49]. The differences in the expression of OBPs and CSPs in different *D. rybakowi* adult tissues were analyzed using SPSS 22.0 software. Paired-sample *t*-tests were used to compare the significant differences in the gene expression of the same tissues between male and female adults (α = 0.05). The significant differences between different tissues of the same sex were analyzed via one-way ANOVA (Duncan’s HSD, α = 0.05). Three biological replications and two technical replications were used for each experiment.

## 3. Results

### 3.1. Antennal Transcriptome and Functional Annotation

The antennal transcriptome involved six samples divided into male and female groups. Trinity v2.14.0 software was used for transcript splicing, and a total of 51,124 unigenes were obtained (Table S1). Gene Ontology (GO) annotations of single genes based on Blastx searches were retrieved from the NR database. A total of 9233 (18.06%) genes were annotated, and the genes expressed in the antennae were mostly associated with binding and catalytic activity in the molecular function category. In the biological processes category, cellular processes, metabolic processes, and biological regulation were the most represented. In the cellular component category, cells, cell parts, and membranes were the most abundant (Figure S1).

### 3.2. Identification of Candidate Reference Genes, OBPs, and CSPs

#### 3.2.1. Candidate Reference Genes

Ten candidate reference genes (*ACT*, *GAPDH*, *TUB*, *RPL13a*, *SYN6*, *RPS18*, *RPL19*, *GST*, *RPS15*, and *EF1a*) were screened out from the *D. rybakowi* antennal transcriptome and were predicted to have full-length ORF-encoding amino acid sequences ranging from 148 to 462 aa. Information on the reference genes can be found in File S3. In addition, high-level similarities found in the Blastx best-hit results with other Coleoptera species ranged from 74.55% to 100.00%, and the species included *Agrilus planipennis*, *Diabrotica virgifera virgifera*, *Anoplophora glabripennis*, and *Sitophilus oryza* (Table 1).

#### 3.2.2. Identification of OBPs

Bioinformatics analysis showed eleven different sequences of *D. rybakowi* encoding OBPs. Sequence analysis showed that these genes demonstrated ORF and predicted signal peptide sequences. All candidate OBP sequences in the Blastx’s best hits were similar to the known OBP genes of *G. daurica* and *C. bowringi*, with their identity ranging from 28.89% to 89.78%. The protein sequences of DrybOBPs ranged from 120 to 220 amino acids (Table 2). In addition, DrybOBP1~DrybOBP9 belong to the minus-C OBP group, containing four conserved cysteines with a Cys1-X_28–32_-Cys2-X_37–39_-Cys3-X_16–23_-Cys4 pattern, in which X signifies any amino acid except for Cys. DrybOBP10 was identified as a classic OBP and had six conserved cysteines, and the general formula was Cys1-X_27_-Cys2-X_3_-Cys3-X_29_-Cys4-X_9_-Cys5-X_8_-Cys6. DrybOBP11, a plus-C OBP, contained eight conserved cysteines, and the following pattern was Cys1-X_35_-Cys2-X_3_-Cys3-X_43_-Cys4-X_13_-Cys4a-X_9_-Cys5-X_8_-Cys6-X_10_-Cys6a (Figure S2).

**Figure 1:**
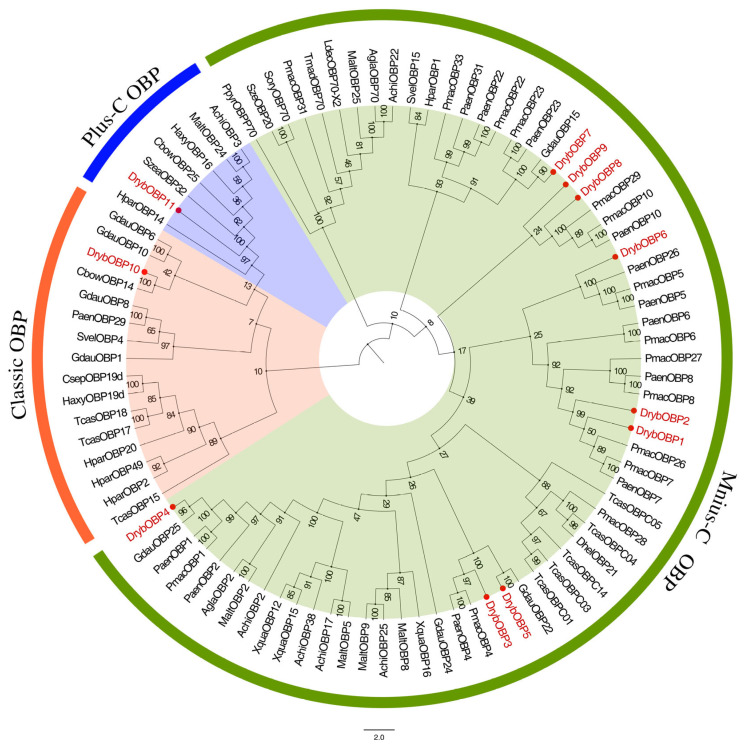
Phylogenetic tree of DrybOBPs with other Coleopteran OBPs. The red circles and red font mark candidate DrybOBPs. Green, orange, and blue represent minus-C OBP, classic OBP, and plus-C OBP genes, respectively. Phylogenetic tree composition information is shown in File S1.

We selected 91 OBP genes from 19 Coleoptera species for OBP phylogenetic analysis, including *G. daurica*, *Holotrichia parallela*, *Pyrrhalta aenescens*, *P. maculicollis*, and *Tribolium castaneum*. Phylogenetic analysis showed that the tree is divided into two branches: minus-C OBPs were clustered into one branch, and plus-C OBPs and classic OBPs were clustered into another. DrybOBPs have a closer kinship and genetic distance to *G. daurica*, *T. castaneum*, and *P. aenescens* (Figure 1). DrybOBP1 and DrybOBP2 were homologs of PmacOBP26, PmacOBP7, and PaenOBP7. DrybOBP3 and DrybOBP5 were closely related to PmacOBP4, PaenOBP4, and GdauOBP22. DrybOBP4 was a homolog of GdauOBP25. DrybOBP6 was closely related to PaenOBP26 and PmacOBP5. DrybOBP7 was closely related to GdauOBP15 and PaenOBP23. DrybOBP8 and DrybOBP9 were closely related to PmacOBP29, PmacOBP10, and PaenOBP10. DrybOBP10 was a homolog of CbowOBP14. DrybOBP11 was a homolog of HparOBP14 (Figure 1).

#### 3.2.3. Identification of CSPs

**Figure 2:**
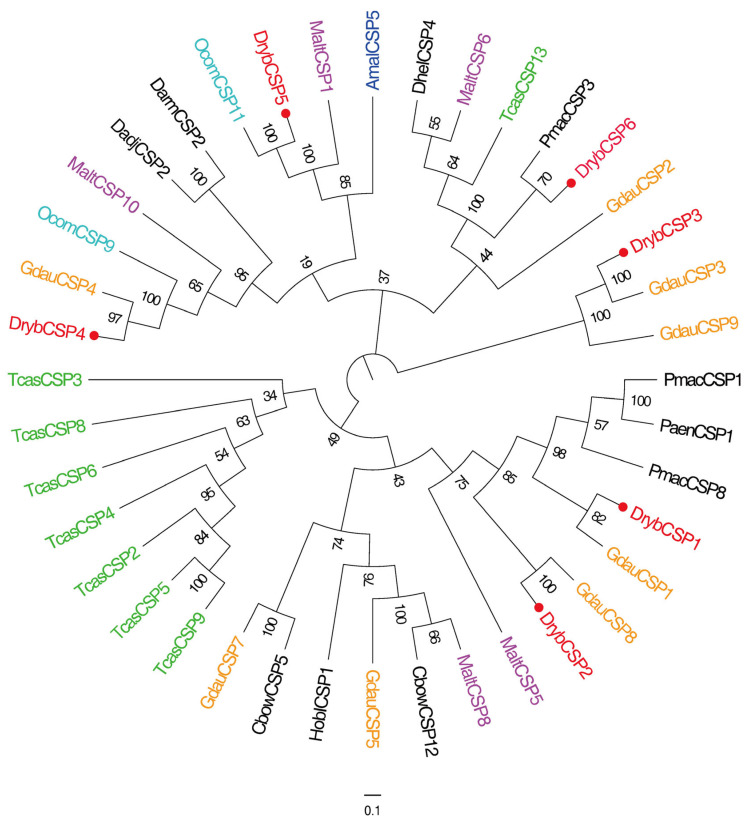
Phylogenetic tree of DrybCSPs with other coleopteran CSPs. The red circles and red font mark candidate DrybCSPs. Representation of the main same species in different color fonts. Phylogenetic tree composition information is shown in File S2.

Six chemosensory protein genes (DrybCSP1~DrybCSP6) were identified from the antennal transcriptome of *D. rybakowi*. These genes consisted of full-length open reading frames (ORFs) encoding 117–232 amino acid residues, and the identities for DrybCSPs comparisons ranged from 65.90% to 93.38% (Table 2). The amino acid sequence alignment showed that six DrybCSP genes had four conserved cysteines and satisfied the sequence structure characterized by Cys1-X_6–8_-Cys2-X_18_-Cys3-X_2_-Cys4 (Figure S3). To show the homologous relationship between DrybCSPs and other Coleoptera insects, we selected 40 CSP genes from 13 Coleoptera species to construct a phylogenetic tree. In terms of species affinities, DrybCSPs were more closely related to *G. daurica* CSPs. Moreover, the phylogenetic tree is divided into three branches. DrybCSP1 and DrybCSP2 were grouped into one branch, DrybCSP3 was grouped into another branch, and DrybCSP4, DrybCSP5, and DrybCSP6 were separated into the third branch. For CSPs, DrybCSP1 was closely related to GdauCSP1. DrybCSP2 was closely related to GdauCSP8 and MatlCSP5. DrybCSP3 were homologs of GdauCSP3 and GdauCSP9. DrybCSP4 was a homolog of GdauCSP4 and OcomCSP9. DrybCSP5 was a homolog of OcomCSP11 and MaltCSP1. DrybCSP6 was closely related to PmacCSP3 (Figure 2).

### 3.3. Validation and Design of RT-qPCR Primers

The primer specificity of ten candidate reference genes was verified by the presence of a single DNA band with the expected product size in 1.5% agarose gel electrophoresis and by a single peak in the RT-qPCR melting curve analyses (Figure S4). Each *D. rybakowi* cDNA was used as a template in RT-qPCR after fivefold serial dilution, and the amplification efficiency (E) of each primer ranged from 97.7% to 131.8%, with associated R^2^ values ranging from 0.92 to 0.99 (Table S2). Meanwhile, the presence of expected-size single DNA bands for OBP and CSP primers was also verified based on the 1.5% agarose gel electrophoresis. All sequence information primers were listed and satisfied the requirements of fluorescence quantitative analysis and were deemed suitable for subsequent quantitative assays (Tables S2 and S3).

### 3.4. Stability of Candidate Reference Genes under Different Conditions

#### 3.4.1. Delta Ct Analysis

**Figure 3:**
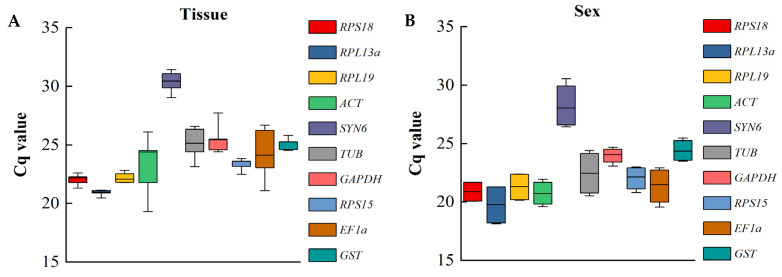
Expression profiles of ten candidate reference genes in four experimental conditions. Each box shows the range of Ct values for each candidate reference gene of *D. rybakowi* under different conditions ((**A**): tissue and (**B**): sex).

The raw Ct values reflected gene stability, with a lower variation in the Ct values indicating more stable genes. In the overall analysis, the average Ct values for ten genes ranged from 18.13 to 31.42, indicating that the expression was high under different experimental conditions and conformed to the reference gene screening criterion. For *RPL13a*, the average Ct values ranged from 20.47 to 21.13, making it the least variable and the most stable gene. The average Ct values of *ACT* ranged from 19.30 to 24.51, demonstrating the highest level of variation and the least stable gene. The raw Ct values of ten candidate reference genes showed similar expression patterns and low concentrated variability, indicating their suitability as reference genes under the different treatment conditions (Figure 3). The reference genes with the highest degree of stability included the following: different tissues (*RPL13a*, Ct = 20.91 ± 0.24) and sexes (*GADPH*, Ct = 23.96 ± 0.61).

#### 3.4.2. GeNorm Analysis

**Figure 4:**
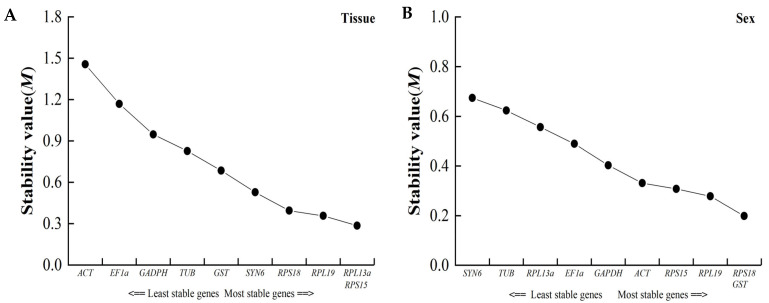
Average expression stability values (M) of candidate reference genes for comparisons of (**A**) different tissues and (**B**) different sexes obtained in the GeNorm software. M value represented the stability of ten candidate reference genes.

In the GeNorm program, we calculated the candidate reference gene stability with the M values. The results showed that all M values for the candidate reference genes ranged from 0.199 to 1.456, making them suitable for use. *RPL13a* and *RPL15*, which had the same values, were the most stable reference genes for different tissues, while *RPLS18* and *GST* were the most stable reference genes for the different sexes (Figure 4).

**Figure 5:**
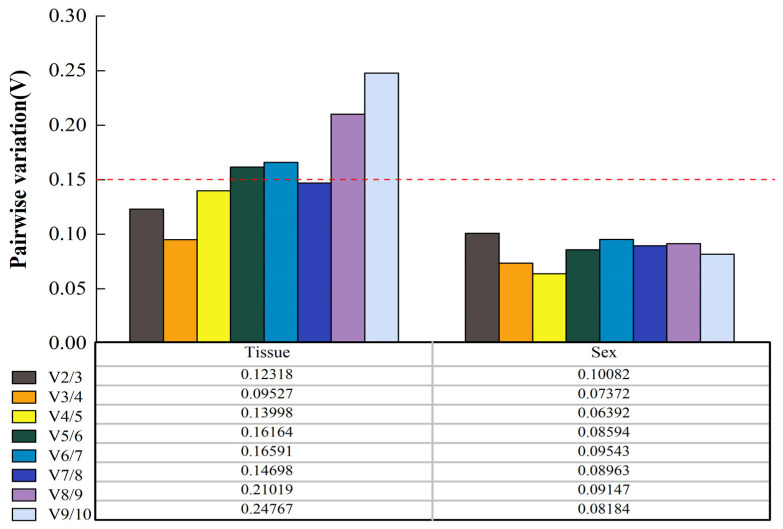
Pairwise variation (V) of candidate reference genes of *D. rybakowi* under two experimental conditions by GeNorm software. The red dotted line represents the critical value of V_n_/V_n+1_.

The GeNorm software also provided an even more important parameter of pairwise variation (V_n_/V_n+1_) to be used in evaluating the optimal number of reference genes under different conditions. (V_n_/V_n+1_) was less than 0.15, and the number of N-candidate reference genes needed to be introduced to correct the relative quantitative data. Based on the above criteria, the V_2/3_ values for the different tissues (V_2/3_ = 0.123) and sexes (V_2/3_ = 0.100), which indicated two optimal reference genes, were enough to accurately normalize different samples (Figure 5).

#### 3.4.3. NormFinder Analysis

The results of the NormFinder analysis showed that the stability under different conditions was determined according to the stability values (SVs) of ten candidate reference genes; the lower the stabilization values, the higher the stability. Concerning stability, the most stable gene was *SYN6* (SV = 0.047) in the different tissues, while the most unstable was *ACT* (SV = 2.457). The rankings of the selected reference genes according to sex were as follows: *RPL19 > ACT > RPS15 > EF1a > RPS18 > GST > RPL13a > TUB > GADPH > SYN6*. (Table 3).

#### 3.4.4. BestKeeper Analysis

The coefficient of variation (CV) and standard deviation (SD) were calculated for the candidate reference genes. A lower SD value means that the gene is more stable in a set of raw Ct values under different conditions, and a lower CV value means that the genes show higher stability. In the different tissue and sex treatments, the most stable genes were *RPL13a* (0.88 ± 0.18) and *GAPDH* (2.25 ± 0.54), respectively, while the most unstable genes were *ACT* (9.22 ± 2.14) and *RPL13a* (7.80 ± 1.54) (Table 4).

#### 3.4.5. RefFinder Analysis

Based on the ranking within each algorithm, appropriate weights were assigned to the individual genes, and the geometric mean of their weights was calculated to arrive at an overall final ranking. The comprehensive ranking of the stability values of ten candidate reference genes in the RefFinder analysis and the optimal number of reference genes determined by combining the pairwise variation (V_n_/V_n+1_) in the GeNorm software identified the optimal reference genes for condition tissues and sexes as *RPL13a* and *RPS18*, and *RPL19* and *GS*, respectively (Table 5).

### 3.5. Relative Gene Expression of OBPs and CSPs

**Figure 6:**
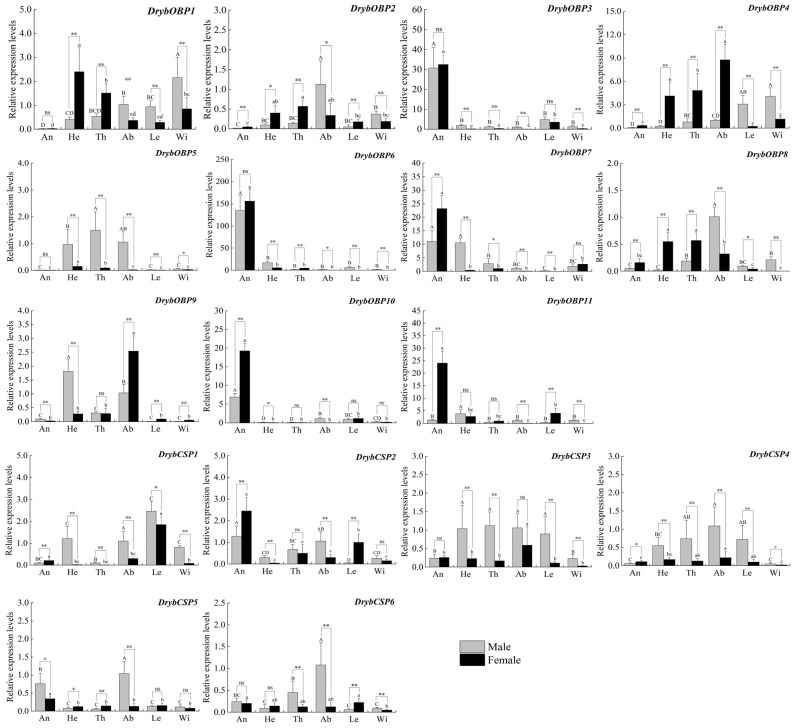
The expression levels of *DrybOBPs* and *DrybCSPs* in different tissues of *D. rybakowi* adult. An: antennae; He: heads; Th: thoraxes; Ab: abdomens; Le: legs; Wi: wings. * indicates significant difference of *DrybOBPs* and *DrybCSPs* between female and male adult (* *p* < 0.05, ** *p* < 0.01); Different upper and lower case letters indicate significant differences of *DrybOBPs* and *DrybCSPs* in different adult tissues for same sexes; ns indicate no significant difference.

As shown in Figure 6, RT-qPCR was used to determine the expression profiles of OBPs and CSPs in different *D. rybakowi* tissues. For OBPs, *DrybOBP3* specifically expressed higher levels in the antennae of the males (F_5,30_ = 47.075, *p* < 0.001) and females (F_5,30_ = 244.334, *p* < 0.001), respectively, than in other tissues. The *DrybOBP3* and *DrybOBP6* expressions were not significantly different between males and females (*DrybOBP3*, t = 0.39, *p* = 0.705; *DrybOBP6*, t = 1.217, *p* = 0.252). *DrybOBP7* and *DrybOBP10* were also abundantly expressed in the antennae, with these genes exhibiting significantly higher expression levels in female antennae than in those of males (*DrybOBP7*, t = 4.644, *p* = 0.001; *DrybOBP10*, t = 113.671, *p* < 0.001). *DrybOBP11* was specifically expressed in female antennae (F_5,30_ = 112.818, *p* < 0.001). For CSPs, *DrybCSP2* exhibited higher expression levels in the antennae of males (F_5,30_ = 17.794, *p* < 0.001) and females (F_5,30_ = 41.820, *p* < 0.001), respectively, than in other tissues. *DrybCSP5* also exhibited high expression levels in the antennae but was significantly higher in males than females (t = 3.27, *p* = 0.005). Additionally, *DrybCSP4*, *DrybCSP5*, and *DrybCSP6* were highly expressed in male abdomens (*DrybCSP4*, F_5,30_ = 8.413, *p* < 0.001; *DrybCSP5*, F_5,30_ = 33.253, *p* < 0.001; *DrybCSP6*, F_5,30_ = 16.852, *p* < 0.001).

## 4. Discussion

### 4.1. Antennal Transcriptome Analysis

With the development and popularization of biotechnology, there have been numerous research reports on olfactory genes in Coleoptera insects, such as *H. axyridis, G. daurica*, and *O. commun*. [11,16,50]. Olfactory genes play an important role in modulation of multiple behaviors, such as locating hosts and foraging mates, searching for egg-laying sites, and avoiding enemies [51,52]. Therefore, developing new strategies, based on insect olfactory perception of chemical substances such as plant volatiles is a major research direction for the future [53]. In this study, eleven candidate OBPs, six CSPs, and ten reference genes for olfactory genes were identified from the antennal transcriptome of the Coleoptera pest *D. rybakowi*. For Coleoptera insects, 25 OBP genes were identified in the antennal transcriptome of *C. bowringi* [12], 26 OBPs in *O. communa* [50], 31 OBPs in *H. axyridis* [11], and 29 OBPs in *G. daurica* [16]. The number of OBPs in *D. rybakowi* was lower than previously reported, but eleven OBPs were found in *Podabrus annulatus*, which was the same as in previous reports [54]. Furthermore, upon comparing CSP numbers in *D. rybakowi* with those in other Coleoptera species, there were differences in twelve CSP genes in *H. axyridis* and *C. bowringi*, respectively [11,12], but similar CSP genes were found in *Hylamorpha elegans* (four CSPs), *A. eugenii* (six CSPs) [14], and *C. maculatus* (seven CSPs) [13,55]. The differences in the number of OBPs and CSPs may be due to the variation in the chemical environment of different species, where these species have evolved for a long time [56]. Furthermore, these differences may also be caused by various sequencing methods and sequencing platform technologies [57], such as differences in RNA extraction quality, RNA breakage, purification recoveries, instrument selections for high-throughput sequencing, and assembly software for transcriptome data [58,59,60]. Moreover, different gene function annotation methods can lead to variations in the number of identified olfactory genes. We primarily identified OBP and CSP genes from the NR, NT, and Swiss-Prot databases according to their sequence similarity. However, the lack of olfactory genes discovered in the Coleoptera species with the same family as *D. rybakowi* could lead to fewer OBP and CSP genes. We also identified OBP and CSP genes based on the conserved sequence fragments for structural domain, which probably led to the incomplete ORF sequences not being identified.

### 4.2. Evaluation of Reference Genes Stability

RT-qPCR has been extensively used in gene expression assays for its rapidity, accuracy, high sensitivity, and high specificity [61]. However, the stability expression of the candidate reference genes under different conditions was not unanimous [28,62]. Therefore, if we wish to improve the reliability of the RT-qPCR experimental results, it is crucial to select the most stable reference genes under different experimental conditions [63]. In recent years, evaluations of optimal reference genes to be used as internal controls for RT-qPCR analysis under different conditions (e.g., sex, developmental, tissues, and RNAi) have been reported in a multitude of Coleoptera species, such as *Ips typographus* [29], *C. maculatus* [30], *Agasicles hygrophila* [33], and *Aquatica leii* [62]. This report has focused on validating the candidate reference genes in *D. rybakowi* for RT-qPCR under different conditions. Based on previous studies, we selected ten candidate reference genes (*ACT*, *GAPDH*, *TUB*, *RPL13a*, *SYN6*, *GST*, *RPS15*, *EF1a*, *RPS18*, and *RPL19*) commonly used in many insects for evaluation [28,32]. The evaluation of five methods, specifically Delta Ct, GeNorm, NormFinder, BestKeeper, and RefFinder, showed that each method recommended different categories of optimal reference genes. In this study, the reference genes recommended via the five methods in different tissues were *RPL13a*, *RPL13a* and *RPL5*, *SYN6*, *RPL13a*, and *RPL13a* and *RPS18*, respectively. The differences in the number of reference genes recommended via each method are due to different computational principles, but RefFinder was developed by synthesizing the principles of four previously reported methods; Therefore, the results are more accurate [33,48]. The *ACT* gene has been widely used as a reference gene for determining tissue expression, such as in *T. castaneum* [32], *C. maculatus* [30], *I. typographus* [29], and *A. leii* [62]. On the contrary, *ACT* was the least stable reference gene in *Phaedon brassicae* [31]. This is similar to our findings showing that *RPL13a* and *RPS18* exhibited good stability for expression in different tissues in *D. rybakowi*, and *ACT* was the least stable reference gene. A study on *C. bowringi* [64] and *O. communa* [65] concluded that *RPL19* presented excellent stability across different sexes. Our results showed that *RPL19* and *GST* were rated as the best combination of reference genes for different sexes. The stability of different reference genes varies in different species; thus, we must be rigorous in evaluating the optimal type and number of reference genes [28].

### 4.3. OBP Gene Identification and Expression Profiling

OBPs are unique olfactory proteins found in high abundance in antennal sensilla lymph and are highly structurally conserved between different insects, playing important roles in their olfaction [66,67]. In total, eleven OBP genes with full-length ORFs were identified in the antennal transcriptome of *D. rybakowi*. Phylogenetic analysis showed that most DrybOBPs clustered with the OBPs of *G. daurica*, *P. aenescens*, and *P. maculicollis*, indicating that OBPs had high sequence homology in closely related species [5,68]. RT-qPCR assays showed that *DrybOBP3*, *DrybOBP6, DrybOBP7, DrybOBP10*, and *DrybOBP11* were specifically expressed in the antennae, indicating that these genes may be involved in chemical communication [7]. *DrybOBP3* and *DrybOBP6* showed no difference between male and female antennae. OcomOBP7 is not differentially expressed in male and female antennae and binds strongly to limonene, a-pinene, and ocimene, the major volatile components of *Andromeda*, suggesting that OcomOBP7 is involved in host localization processes [15]. SvelOBP15 is not differentially expressed in either sex and has a high binding affinity for linalool, nerolidol, and limonene, suggesting that it exerts its olfactory function by binding transported plant volatiles [69]. Similarly, RproOBP6 and RproOBP13 were expressed in both male and female antennae, indicating that they probably participate in behaviors common to both sexes, such as host localization and the avoidance of natural enemies [20]. It is hypothesized that *DrybOBP3* and *DrybOBP6* probably have the same function. Furthermore, the *DrybOBP7*, *DrybOBP10*, and *DrybOBP11* expression were significantly higher in female antennae than male antennae. A study demonstrated that HoblOBP7 is specifically expressed in female antennae and strongly binds to dibutyl phthalate, and the response of females is significantly reduced after RNA interference [17]. Dibutyl phthalate was proven to be a major substance in increasing the mating rate [70]. It is hypothesized that *DrybOBP7*, *DrybOBP10*, and *DrybOBP11* have the same function in mating behavior.

### 4.4. CSP Gene Identification and Expression Profiling

CSPs are another class of soluble proteins that bind and translocate chemical molecules in sensillum lymph and are abundantly expressed [71]. We identified six genes encoding chemosensory proteins. Multiple comparisons of CSPs in most Coleoptera species showed that they included four conserved cysteine residues, the same as DrybCSPs, implying that CSPs are highly conserved proteins among insects [15,72]. RT-qPCR assays showed that among the six CSPs, only *DrybCSP2* was highly expressed in antennae. CSPs generally function like OBPs, CSPs function generally similar to OBP, and most of the CSPs highly expressed in the antennae bind strongly to host volatiles and perform important functions in host localization [73,74]. Furthermore, *DrybCSP4*, *DrybCSP5*, and *DrybCSP6* were highly expressed in the male abdomen. However, AlepCSP2 exhibits strong binding with these two sex pheromones (Z7-12: Ac and Z9-14: Ac), and the antennal electrical response of siCSP2 males is significantly decreased, and the mating rate is significantly decreased by 37.50% [22]. AmalCSP5 was abundantly expressed in the male abdomen. It exhibited strong binding affinity for the host volatile of apple trees (hexyl benzoate and hexyl hexanoate) and secondary compounds (phlorizin, kaempferol, chlorogenic acid, and rutin), suggesting that AmalCSP5 may play an essential role in olfactory responses of beetles (e.g., choice of host plants and oviposition sites), and also play a role in repelling pests via gustation and contact chemoreception [74]. It is hypothesized that *DrybCSP4*, *DrybCSP5*, and *DrybCSP6* have similar functions in recognizing sex pheromones and in the mating process. However, these hypothesized gene functions must be further validated via experiments such as fluorescent competitive binding assays, RNA interference, and CRISPR/Cas9.

## 5. Conclusions

We screened and identified OBPs, CSPs, and suitable reference genes from the *D. rybakowi* antennal transcriptome. Firstly, we followed a standardized RT-qPCR procedure, and two reference genes were used to correct the quantitative data under two conditions. The recommendations are as follows: tissues (*RPL13a* and *RPS18*) and sexes (*RPL19* and *GST*. Secondly, we found that *DrybOBP3*, *DrybOBP6*, *DrybOBP7*, *DrybOBP10*, *DrybOBP11, DrybCSP2*, and *DrybCSP5* are more abundantly expressed in the antennae than in other tissues. This means that these genes are target genes for the development of green chemical attractants or repellents using olfactory functions to control the *D. rybakowi* leaf beetle. Subsequently, we will use fluorescence competition binding tests, electroantennography, and behavioral response experiments to further study the relationship between olfactory proteins and volatile compounds in host plants.

## Figures and Tables

**Table 1:** NCBI Blastx results of ten candidate reference genes in *D. rybakowi*.

Gene_ID	Gene Abbr	Genbank Number	ORF (aa)	Blastx Annotation	Acc. Number	E-Value	Identity (%)
TRINITY_DN13598_c2_g3	*ACT*	OR797776	376	actin, muscle [*Agrilus planipennis*]	XP_018335426.1	0	99.73
TRINITY_DN13848_c0_g3	*GAPDH*	OR797777	332	glyceraldehyde-3-phosphate dehydrogenase 2-like [*Diabrotica virgifera virgifera*]	XP_028133871.1	0	93.66
TRINITY_DN13241_c1_g2	*EF1a*	OR797778	462	elongation factor A [*Diabrotica undecimpunctata howardi*]	APQ43052.1	0	97.77
TRINITY_DN8410_c0_g1	*TUB*	OR797779	447	tubulin beta-1 chain [*Anoplophora glabripennis*]	XP_018568298.1	0	100.00
TRINITY_DN10540_c0_g1	*RPL13a*	OR797780	204	60S ribosomal protein L13a [*Sitophilus oryzae*]	XP_030748989.1	3.00 × 10^−126^	94.61
TRINITY_DN14590_c0_g1	*RPS18*	OR797781	152	ribosomal protein S18 [*Phaedon cochleariae*]	AFQ22730.1	5.00 × 10^−89^	99.23
TRINITY_DN10601_c0_g1	*RPL19*	OR797782	199	ribosomal protein L19 [*Chrysomela tremula*]	ACY71295.1	3.00 × 10^−136^	97.49
TRINITY_DN14218_c0_g1	*SYN6*	OR797783	153	syntaxin-6 [*Anoplophora glabripennis*]	XP_018565804.1	5.00 × 10^−84^	92.54
TRINITY_DN1008_c0_g1	*GST*	OR797784	220	glutathione S-transferase 1 [*Anoplophora glabripennis*]	XP_018568551.1	4.00 × 10^−118^	75.45
TRINITY_DN11144_c0_g1	*RPS15*	OR797785	148	ribosomal protein S15 [*Stegobium paniceum*]	ALG76024.1	2.00 × 10^−76^	94.59

**Table 2:** Summary of candidate OBPs and CSPs identified in *D. rybakowi*.

Gene_ID	Gene Name	Genebank Number	Complete ORF (aa)	Signal Peptide	Group	BLAST Annotation	Acc. Number	E-Value	Identity (%)
**Odorant-binding proteins, OBPs**									
TRINITY_DN10570_c0_g1	*DrybOBP1*	OR797799	126	1-16	Minus-C OBP	odorant-binding protein 26 [*Pyrrhalta maculicollis*]	APC94196.1	2.00 × 10^−25^	51.92
TRINITY_DN10570_c0_g2	*DrybOBP2*	OR797786	130	1-16	Minus-C OBP	odorant-binding protein 26 [*Pyrrhalta maculicollis*]	APC94196.1	4.00 × 10^−38^	51.64
TRINITY_DN10673_c0_g1	*DrybOBP3*	OR797787	143	1-17	Minus-C OBP	odorant-binding protein [*Galeruca daurica*]	AQY18990.1	7.00 × 10^−58^	65.6
TRINITY_DN12287_c4_g1	*DrybOBP4*	OR797800	137	1-16	Minus-C OBP	odorant-binding protein [*Galeruca daurica*]	AQY18989.1	1.00 × 10^−86^	89.78
TRINITY_DN12797_c0_g5	*DrybOBP5*	OR797788	135	1-18	Minus-C OBP	odorant-binding protein [*Galeruca daurica*]	AQY18986.1	1.00 × 10^−66^	77.1
TRINITY_DN12797_c0_g7	*DrybOBP6*	OR797789	134	1-18	Minus-C OBP	odorant-binding protein 5 [*Pyrrhalta maculicollis*]	APC94193.1	9.00 × 10^−35^	46.32
TRINITY_DN14706_c2_g1	*DrybOBP7*	OR797790	167	1-17	Minus-C OBP	odorant-binding protein [*Galeruca daurica*]	AQY18987.1	1.00 × 10^−58^	63.64
TRINITY_DN14781_c6_g3	*DrybOBP8*	OR797791	130	1-17	Minus-C OBP	odorant-binding protein 29 [*Pyrrhalta maculicollis*]	APC94190.1	4.00 × 10^−51^	64.12
TRINITY_DN29911_c0_g1	*DrybOBP9*	OR797792	136	1-16	Minus-C OBP	odorant-binding protein [*Galeruca daurica*]	AQY18985.1	1.00 × 10^−11^	28.89
TRINITY_DN14470_c4_g1	*DrybOBP10*	OR797801	120	1-23	Classic-OBP	odorant-binding protein [*Galeruca daurica*]	AQY18968.1	4.00 × 10^−61^	77.5
TRINITY_DN11053_c0_g1	*DrybOBP11*	OR797793	220	0	Plus-c OBP	odorant-binding protein 25 [*Colaphellus bowringi*]	ALR72513.1	2.00 × 10^−53^	45.81
**Chemosensory proteins, CSPs**									
TRINITY_DN11935_c0_g1	*DrybCSP1*	OR797794	129	1-18	/	chemosensory protein	ARM20137.1	2.00 × 10^−51^	79.84
TRINITY_DN13680_c2_g1	*DrybCSP2*	OR797795	131	1-19	/	ejaculatory bulb-specific protein 3-like [*Diorhabda carinulata*]	XP_057656967.1	3.00 × 10^−57^	91.89
TRINITY_DN14709_c1_g3	*DrybCSP3*	OR797796	136	1-21	/	chemosensory protein	ARM20139.1	4.00 × 10^−76^	93.38
						[*Galeruca daurica*]			
TRINITY_DN14908_c1_g2	*DrybCSP4*	OR797797	124	1-22	/	ejaculatory bulb-specific protein 3-like	XP_056639215.1	3.00 × 10^−79^	91.87
TRINITY_DN8389_c0_g1	*DrybCSP5*	OR797802	117	1-22	/	chemosensory protein 11	UMT69263.1	5.00 × 10^−64^	83.9
						[*Ophraella communa*]			
TRINITY_DN9992_c0_g1	*DrybCSP6*	OR797798	232	1-18	/	chemosensory protein	ARM20146.1	2.00 × 10^−82^	65.9
						[*Galeruca daurica*]			
TRINITY_DN11935_c0_g1	DrybCSP1	OR797794	129	1-18	/	chemosensory protein	ARM20137.1	2.00 × 10^−51^	79.84
						[*Galeruca daurica*]			
TRINITY_DN13680_c2_g1	DrybCSP2	OR797795	131	1-19	/	ejaculatory bulb-specific protein 3-like [*Diorhabda carinulata*]	XP_057656967.1	3.00 × 10^−57^	91.89
TRINITY_DN14709_c1_g3	DrybCSP3	OR797796	136	1-21	/	chemosensory protein	ARM20139.1	4.00 × 10^−76^	93.38
						[*Galeruca daurica*]			
TRINITY_DN14908_c1_g2	DrybCSP4	OR797797	124	1-22	/	ejaculatory bulb-specific protein 3-like	XP_056639215.1	3.00 × 10^−79^	91.87
						[*Diorhabda carinulata*]			
TRINITY_DN8389_c0_g1	DrybCSP5	OR797802	117	1-22	/	chemosensory protein 11	UMT69263.1	5.00 × 10^−64^	83.9
						[*Ophraella communa*]			
TRINITY_DN9992_c0_g1	DrybCSP6	OR797798	232	1-18	/	chemosensory protein	ARM20146.1	2.00 × 10^−82^	65.9
						[*Galeruca daurica*]			

**Table 3:** Ranking of ten candidate reference genes under different conditions by NormFinder software in *D. rybakowi*.

Rank	Tissue		Sex	
	Gene	SV	Gene	SV
1	*SYN6*	0.047	*RPL19*	0.169
2	*RPS18*	0.125	*ACT*	0.249
3	*RPL19*	0.583	*RPS15*	0.322
4	*RPL13a*	0.659	*EF1a*	0.346
5	*TUB*	0.755	*RPS18*	0.445
6	*RPS15*	0.815	*GST*	0.495
7	*GAPDH*	0.834	*RPL13a*	0.507
8	*GST*	1.347	*TUB*	0.71
9	*EF1a*	1.816	*GADPH*	0.775
10	*ACT*	2.457	*SYN6*	0.802

**Table 4:** Stability analysis of ten candidate reference genes by BestKeeper software in *D. rybakowi*.

Rank	1	2	3	4	5	6	7	8	9	10
	CV ± SD	CV ± SD	CV ± SD	CV ± SD	CV ± SD	CV ± SD	CV ± SD	CV ± SD	CV ± SD	CV ± SD
Tissue	*RPL13a*	*GST*	*RPL19*	*RPS18*	*RPS15*	*SYN6*	*GAPDH*	*TUB*	*EF1a*	*ACT*
	0.88 ± 0.18	1.68 ± 0.42	1.72 ± 0.38	1.76 ± 0.39	1.76 ± 0.41	2.41 ± 0.73	3.44 ± 0.88	4.27 ± 1.07	7.36 ± 1.78	9.22 ± 2.14
Sex	*GAPDH*	*GST*	*RPS18*	*RPS15*	*ACT*	*RPL19*	*SYN6*	*EF1a*	*TUB*	*RPL13a*
	2.25 ± 0.54	3.48 ± 0.85	3.82 ± 0.8	4.11 ± 0.91	4.46 ± 0.93	5.07 ± 1.08	5.91 ± 1.67	6.44 ± 1.38	7.55 ± 1.7	7.80 ± 1.54

**Table 5:** Overall stability ranks of ten candidate reference genes by five methods in *D. rybakowi*.

Conditions	Reference Gene	RefFinder		ΔCt		GeNorm		NormFinder		BestKeeper		Recommendation
		Stability	Rank	Stability	Rank	Stability	Rank	Stability	Rank	Stability	Rank	
**Tissue**												
	*ACT*	10.000	10	2.600	10	1.455	9	2.457	10	2.140	10	*RPL13a*, *RPS18*
	*TUB*	6.400	6	1.320	6	0.827	6	0.755	5	1.074	8	
	*RPS18*	2.210	2	1.030	1	0.394	3	0.125	2	0.388	3	
	*GST*	6.620	7	1.600	8	0.685	5	1.347	8	0.422	5	
	*SYN6*	2.780	3	1.090	2	0.527	4	0.047	1	0.732	6	
	*GAPDH*	7.240	8	1.440	7	0.947	7	0.834	7	0.879	7	
	*EF1a*	9.000	9	2.040	9	1.168	8	1.816	9	1.783	9	
	*RPL13a*	1.860	1	1.120	3	0.285	1	0.659	4	0.163	1	
	*RPL19*	2.910	4	1.130	4	0.356	2	0.583	3	0.381	2	
	*RPL15*	3.310	5	1.200	5	0.285	1	0.815	6	0.412	4	
**Sex**												
	*ACT*	3.160	3	0.560	2	0.331	4	0.249	2	0.926	5	*RPL19*, *GST*
	*TUB*	8.710	9	0.790	8	0.623	8	0.710	8	1.696	9	
	*RPS18*	2.510	2	0.610	4	0.199	1	0.445	5	0.797	2	
	*GST*	3.220	4	0.650	6	0.199	1	0.495	6	0.849	3	
	*SYN6*	9.740	10	0.880	10	0.674	9	0.802	10	1.670	9	
	*GAPDH*	4.700	6	0.840	9	0.403	5	0.775	9	0.539	1	
	*EF1a*	5.600	7	0.620	5	0.489	6	0.346	4	1.376	7	
	*RPL13a*	7.480	8	0.680	7	0.557	7	0.507	7	1.540	8	
	*RPL19*	2.060	1	0.540	1	0.279	2	0.169	1	1.079	6	
	*RPL15*	3.460	5	0.570	3	0.308	3	0.322	3	0.905	4	

## Data Availability

The data presented in this study are deposited in the Figshare repository, accession number https://figshare.com/s/dcdc3a314678f4dbf236 (accessed on 3 February 2024).

## Supplementary Materials

The following supporting information can be downloaded at https://www.mdpi.com/article/10.3390/insects15040251/s1, Figure S1: Gene ontology (GO) assignment of *D. rybakowi* unigenes. Figure S2: Sequence alignment of OBPs in *D. rybakowi*. Figure S3: Sequence alignment of CSPs in *D. rybakowi*. Figure S4: Primer validation and melting curves of ten candidate reference genes. Table S1: Assembly summary of antennal transcriptome in *D. rybakowi*. Table S2: Primer sequences, product sizes, and PCR efficiencies of ten candidate reference genes in *D. rybakowi*. Table S3: Primer used in RT-qPCR of DrybOBPs and DrybCSPs. File S1: It includes the amino acid and nucleic acid sequences of DrybOBPs, as well as the amino acid sequences and GenBank numbers of OBPs of other Coleoptera insects in the phylogenetic tree. File S2: It includes the amino acid and nucleic acid sequences of DrybCSPs, as well as the amino acid sequences and Genbank numbers of the CSPs of other Coleoptera insects in the phylogenetic tree. File S3: It includes the amino acid and nucleic acid sequences of reference genes and expression data for the reference genes in different tissues and sexes.
