# Supplementary material for: Antennal Transcriptome Evaluation and Analysis for Odorant-Binding Proteins, Chemosensory Proteins, and Suitable Reference Genes in the Leaf Beetle Pest Diorhabda rybakowi Weise (Coleoptera: Chrysomelidae)
Source: Insects. 2024 Apr 7;15(4):251. doi: 10.3390/insects15040251 (PMC11050234; doi:10.3390/insects15040251)
Supplement: Supplementary file 1 [file insects-15-00251-s001.zip › Appendix A.pdf]

Phylogenetic tree of DrybOBPs

>OR797799 DrybOBP1 odorant binding protein 1, complete cds [Diorhabda rybakowi Weise]

ATGAAGACTGTTCTAGTAATTACTCTGTTGATTGCCGTAACTCTGGCTGGTCAACGTAAAGATGAAATACAAG
CAGCTGTCAAAGCCTGTGATGGTGAATACGAAGGACCGAATGATTTAAGAAAATTAGTTATGGAAAAAGAT
GCTGACAAAGTCAAATTGGGCGAAAAAATATTTTGCATCAACAAGAAAATTGGGTTGCAAAAAGAAAATGG
GGATATCGATGAAGAAGTATTCATAAAAGATTCGGAGCATATCAAAGATGACATTTTCAAAGAGAAAGTGAA
AGTATGTATGAAACGAGATGGGGTCACTCCAGAAGAAGCTGCTTTTAACTACTTGAAATGTGTTCAATCTACT
TTACAATCAGTTCATGCATAG

> DrybOBP1

MKTVLVITLLIAVTLAGQRKDEIQAAVKACDGEYEGPNDLRKLVMEKDADKVKLGEKIFCINKKIGLQKENGDID
EEVFIKDSEHIKDDIFKEKVKVCMKRDGVTPEEAAFNYLKCVQSTLQSVHA

>OR797786 DrybOBP2 odorant binding protein 2, complete cds [Diorhabda rybakowi Weise]

ATGAAGGTTGTTTTCATATTTTCTCTGGTTATTTCCATATCTTTGGCAAATTCCATAGAAGAGATAAAAGAATT
GAAAGCGGCTTACAAAGAATGCGGTGGTGAATTCGAAGGGCCAAATAGTGTGAAAGCACAAGTTTTAAAA
GAAAATGTGGACAAAGTTAAATTGGGCGAAACTATTTTTTGTATCAATAAGAAGATAGGTTTGCAAAATGAT
AATGGAAACATCGATGAACAAATTTTCAGAAAATCCCTGCAATTTAAGCTTTCAGACGAATCTTTGATTAACA
AAGTAGCCAAAGAATGTTCTAAACGTGAAGGTGATACTAATGAAGTTATAGCTTTTAATTTTTTGAAATGTTAT
CAATTTATGATTAAGAATAATTTGAACTAG

>DrybOBP2

MKVVFIFSLVISISLANSIEEIKELKAAYKECGGEFEGPNSVKAQVLKENVDKVKLGETIFCINKKIGLQNDNGNIDE
QIFRKSLQFKLSDESLINKVAKECSKREGDTNEVIAFNFLKCYQFMIKNNLN

>OR797787 DrybOBP3 odorant binding protein 3, complete cds [Diorhabda rybakowi Weise]

ATGCACTTCTCCGTATTTGCTTTATTTTTTGTTTTCGCTGTGGTATCAGCCGAAATAAGTAACGAACCAGAAGA
ATCCTTCAAGAAAATACATGACCAATGCCAATTAGACCCCGCTTCACAAGTAAATGAAGCTATTTTCGATAAA
ATAAGACATGGTGAGAAAGTCACCGATCCCAAATTGGCAAAACACGCTCTATGTATGAACGTAGGACTTGGA
GTTCAAAACCAAAATGGAGACATCAACGTAGATAAACTCAGAGAATCTATTGAGAAATTTACTGATAACAAA
GAAAAAGTTGACGAAGTTGTTAATAAATGTGGCACCAGAACTTCTACGAACGCTGAAGAAGCTGCTGTTGG
TTTGTCAGATTGTATCATGAGCTTCAGACCTCCCTCACCTCAAGGCGAACATCATGGTCATCATCACTAG

>DrybOBP3

MHFSVFALFFVFAVVSAEISNEPEESFKKIHDQCQLDPASQVNEAIFDKIRHGEKVTDPKLAKHALCMNVGLGV
QNQNGDINVDKLRESIEKFTDNKEKVDEVVNKCGTRTSTNAEEAAVGLSDCIMSFRPPSPQGEHHGHHH

>OR797800 DrybOBP4 odorant binding protein 4, complete cds [Diorhabda rybakowi Weise]

ATGAAGATTGTACTCGTATTTTTGTGTGCTCTTGTTTCTGTTTTGGCACGTGGTTTGCCAAACCTTCCAGAAC
ACGAACGCATAAAACTAGCTGAAGTCCACCGGTCTTGTCAATCCAATCCAAAAACATTCTGCGACGAAAACA
AATTGAAGAATCTTTTCAATAACATCAATGACCCTCAAGTCGGCACTCACATGCTGTGTATGGCAGTCAAAGC
AGGATTGATGAGACCAAATGGGGATTTCGATATTTCCTATTTGAAGCAGAAAGTTAGTTTGGTAGTACGCGA
TCATTCTAAAGCAGAGGAACTTGTTCAGAAGTGTACTCGTAAAGGAGAAAACCCAGGAAAAACTGCCAATT
TGATGTGGAAATGTTTGGTTGAAAATGATGTTCAGTATTATCATCAGTTGTAA

>DrybOBP4

MKIVLVFLCALVSVLARGLPNLPEHERIKLAEVHRSCQSNPKTFCDENKLKNLFNNINDPQVGTHMLCMAVKA
GLMRPNGDFDISYLKQKVSLVVRDHSKAEELVQKCTRKGENPGKTANLMWKCLVENDVQYYHQL

>OR797788 DrybOBP5 odorant binding protein 5, complete cds [Diorhabda rybakowi Weise]

ATGAAAATCGCAACAGTGATATGGTTCTTCGCAATTTTTGCAATGGTATCAGCAGTAGCACCACCTGGCCGAT
TCAAAGTAGCACATGATAAATGCCAATCTGATCCAGCATCGTACGTCGATGAATCGATTCTAGAGAAAGTAAG

ACGTGGCGAAAAAGTTACTGATCCCAATCTGGCGAAACATACTCTTGTATGAACGTGAAAACTGGTGTTCA
GAGCCAAAATGGAGACATAATCGTGGAAAATCTCAAAAAATTCCTCGAGAGTGCAGGAAATAGTAAAGAGA
AAGTGGACGAAGCTATCAGCAAATGTGGTACCAGGGCTTCCGCTAACGCAGATGAAGCTGCCGTAGCTTTG
ACAAACTGTGTTATAAAATTTAGACCAGAACGCCCTCATCATCATTGA

>DrybOBP5

MKIATVIWFFAIFAMVSAVAPPGRFKVAHDKCQSDPASYVDESILEKVRRGEKVTDPNLAKHTLCMNVKTGVQ
SQNGDIIVENLKKFLESAGNSKEKVDEAISKCGTRASANADEAAVALTNCVIKFRPERPHHH

>OR797789 DrybOBP6 odorant binding protein 6, complete cds [Diorhabda rybakowi Weise]

ATGAAAATCGCAACAGTGATATGGTTCTTCGCAATTTTTGCAATGGTTTGTGCTGATGATTACATATTATTCTAC
TCAAAACAATTCGCAAGTTCTATACAATGCAACATATTTATGAAAGATATTAATCTAATAAAAGCTAAAACTGA
CACACCATCCAATGTAGATCAATTAGCAAGCAGATTACTGTGTATGAATCAAAAGCTTGGAATGCAAAACGA
ACAGGGTGACTACGTTCCAGCGGAAATCAGAAATTATTTTGAACATGTAGTTGTCAACAAAGATAACATTGA
AAAAATTGTGAATCACTGTTCAGTTAAATCTGATGGAACTACAGCTAAAGAAGCTGCTCTGAATTTATTTGAT
TGTGCCAAGAAGATAGATTCTTTACGATTATCCTCTTGA

>DrybOBP6

MKIATVIWFFAIFAMVCADDYILFYSKQFASSIQCNIFMKDINLIKAKTDTPSNVDQLASRLLCMNQKLGMQNE
QGDYVPAEIRNYFEHVVVNKDNIEKIVNHCSVKSDGTTAKEAALNLFDCAKKIDSLRLSS

>OR797790 DrybOBP7 odorant binding protein 7, complete cds [Diorhabda rybakowi Weise]

ATGAACAAACTATTTCTATTCGCAGTACTCTGCATCGGTGTTGCCAACAGCGAATTGGCTGAATCGGAGTATG
GTACCGAACTGCTTAAGTTGGCAAAAGAGACTCACGATAAGTGTATTGAGCAAACTGGTGTGACACAAGCG
GCAATGAATGAAGTGAAACAAGGTAAATTTGATGATGATGAAAAAATGAAGGAATACATCACCTGTCTGTGG
GTACAGGGCAAAGGGCTGAATGAAAAATTTGAGCTCAATACGGAATTAATAAGAAATGTGTTACCAGAGAA
AATCAAAGACGTGGAATTGAAAGTTATGATGGAGTGCCATGACGAAATACAAGCAAAAGCTGGAGCCGATC
CCAGGTTCGTAGATAAGACGTACTCACTCGCCAAATGTGTCTACAATAAGAAGCCAGAGGTGAGTAATTTTG
GAGTCTTCATTCATTTCGCCAACAAAGCCTTCACTACCATTTTTTCTTTCAACGCCAATATAATTATTAAATGA

>DrybOBP7

MNKLFLFAVLCIGVANSELAESEYGTELLKLAKETHDKCIEQTGVTQAAMNEVKQGKFDDDEKMKEYITCLWVQ
GKGLNEKFELNTELIRNVLPEKIKDVELKVMMECHDEIQAKAGADPRFVDKTYSLAKCVYNKKPEVSNFGVFIHF
ANKAFTTIFSFNANIIIK

>OR797791 DrybOBP8 odorant binding protein 8, complete cds [Diorhabda rybakowi Weise]

ATGATGTGTCTAAGAATAGTATTGTGTGTTTGTCTGGCTATAAGTAACGCTAAGCCTATTTCGGATGACTTACA
AAAGATGCAAAAGAGTCACGAAGAATGTATAAAAGAAGTTGGAATAGAAAAAGTCGATTTGGATAAAGTTT
TAGAATCGAATTTGCCTGAAAGAACGTTGGAAAAATACTCTTATTGCTACGGTAAAAAATTCGGATACATCGG
GGCAGATAATAAAATCGATAGAATTATTGCTAAAGACCAATTCGAAAAAATCATCCCTGGAACATCTGAGGAA
TTCATGAAAGTGTGCATACCCGAAGGAGAGGTAACTGAAAAAGTAGCCTTGGGAATACAACGGTGTATCGG
TCAAAAAACTAAAGAGTTATCAAAACAATAA

>DrybOBP8

MMCLRIVLCVCLAISNAKPISDDLQKMQKSHEECIKEVGIEKVDLDKVLESNLPERTLEKYSYCYGKKFGYIGADN
KIDRIIAKDQFEKIIPGTSEEFMKVCIPEGEVTEKVALGIQRCIGQKTKELSKQ

>OR797792 DrybOBP9 odorant binding protein 9, complete cds [Diorhabda rybakowi Weise]

ATGAAAGAAGTTTTCGTATTTCTATGTCTAGTATCGTGTATTTGGGGTATGCGGTTGCCACCATCAGAGATAAA
AGAATTGAACAAAGTTCATCACGAATGTTTGATTAAAACTAAATGTGATAGAAAATACGTTGCTCATCGTTAT
GAATACCTTGGTTACGAACCTTTGAGAGAATATTTGTTATGTCTTGCAACACGTTATGGTTTGATGTATCCCTC
TGGAGATTTCAACTACGAAACATTGGAATATAAATTTCTGATAATTTGCGAGAAGGGAGAAGATCCTGAATAT

TTTATAAAAAGATGTGCTCGACCTTCTTCTATTTCCGATGCTCGTCAAATAGCAGTTGACTTTTGGACGTGCTT
AGAACAAATAAATTTTCATTATTTGAGTTTTATTAATATATAA

>DrybOBP9

MKEVFVFLCLVSCIWGMRLPPSEIKELNKVHHECLIKTKCDRKYVAHRYEYLGYEPLREYLLCLATRYGLMYPSGD
FNYETLEYKFLIICEKGEDPEYFIKRCARPSSISDARQIAVDFWTCLEQINFHYLSFINI

>OR797801 DrybOBP10odorant binding protein 10, complete cds [Diorhabda rybakowi Weise]

ATGAATCAAAGTTACTTTGTATTTTATCTCTTGGTCATCATAGCAAGTATCGTACTAGAAGGAAATTGCGCTCC
TTCGGACAACGCTAATGCTACTGTCAAAGATTTTTGTATTAAGGAAACGGGTATTAGTGAAGATAAAGTGAA
GCAAATGGAAGATGAATCTGTAGAAGAATTAGACGAAGATTGTTACTGTTACCTTCATTGTATCTTCATATCTC
TCAAGTTAATTGACGAAAAAGGTAAACTTGATGTGATGGAAACTATGAAGGAATTTCCTGATTTTAACAAAG
AATGTTTAGAAAAAGTTCCTCATATTTTGGAGTGTACTGATATAGCAGCCCTCGATAATTGCGAGTCCTAA

>DrybOBP10

MNQSYFVFYLLVIIASIVLEGNCAPSDNANATVKDFCIKETGISEDKVKQMEDESVEELDEDCYCYLHCIFISLKLID
EKGKLDVMETMKEFPDFNKECLEKVPHILECTDIAALDNCES

>OR797793 DrybOBP11 odorant binding protein 11, complete cds [Diorhabda rybakowi Weise]

ATGTTGGCCAACGATTTGGAAGAACTTTACATCAATTCTCACTTCGTAAACATAAGAAATAGGAGAGATACTT
CGAATGAAATCGAAGGCAAATGTGCGAAAAAAGGAAGCAAACCTTGTTGTGATGACACGTCTTTGAAAAA
GATACACAAAGAACAAAGAGATTACAAGGAAGCATGCTTTAAAGAGATAGTAGGAGAAGATAAGCCGGAA
AAACGTCCACATGAAGCACCATTCGATCCTTTCGACTGCGAAAAAATGCGACAAAGAAGAAGTGAGATTGT
GTGCATTTCTCAATGTGTGGGTCAAAAGAATAACTATATTGACGATGACGGTAAGATTAAACCTGATGAATTT
GGAGAATACATAAGAGATCTATTGGAAGATATCACGTATTTAACTCCTGTAGTAGAGAAGATAATTGAAGGAT
GTTTGACCGATATCAAAAATGCTACGATTAAATATGATAAATGTAAACAAGATGGAATGATATCAGAATTTTGT
ATATTTAAAAATATTCAACTGAATTGTCCCGATGATCAAATGAAGAATAAAGAAGCTTGTAAGAAGTTTCAAA
AAATGTTAACAGAAGGTCCACCTGCACTACGTGGCCTACCACCACCAGGTTTTGATGGACCACCACCATCTC
ATTTTCTTGATTAG

>DrybOBP11

MLANDLEELYINSHFVNIRNRRDTSNEIEGKCAKKGSKPCCDDTSLKKIHKEQRDYKEACFKEIVGEDKPEKRPHE
APFDPFDCEKMRQRRSEIVCISQCVGQKNNYIDDDGKIKPDEFGEYIRDLLEDITYLTPVVEKIIEGCLTDIKNATIK
YDKCKQDGMISEFCIFKNIQLNCPDDQMKNKEACKKFQKMLTEGPPALRGLPPPGFDGPPPSHFLD

Phylogenetic tree of Coleopteran OBP sequences

>AUF72964.1 AchiOBP17 odorant-binding protein 17 [Anoplophora chinensis]

MKTALVLLSVIAGTLAASLSEEEKKLQEIHDRCQADPATYVDHELLHHLADNIDNPKVGAHMLCESKAVGLQKP
NGELDLNVIKQKISLTVSDKAKVERLVRECAVKKQTPEKTAVNLFMCLDKDGVTYFHEF

>AUF72969.1 AchiOBP22 odorant-binding protein22 [Anoplophora chinensis]

MFPSVVCVVGSYLIFLTIIQAKEISSGRCDIPPSAPKKVEEIINQCQDEIKLAILSEALQAFNVNEHSRSRAKRATFSE
DEKRIAGCLLQCVYRKMDAVNEKGFPTVQGLVSLYTEGISQKDYILATVQAVNTCLIKSQKKYLITPQSIDENGKT
CDIAYDVFDCVSDEIGKYCGQTP

>AUF72949.1 AchiOBP2 odorant-binding protein2 [Anoplophora chinensis]

MKTLFVFLCVAILGAVAHHLPETEMRKLKEVHDSCQANPTTHVDEKLLKKLKENVEDKKVGTHLLCMAVKVGLL
TQDGDLIKNVIRDKIVIATHDLSKVDEVLKKCAVKRETPEKTAVQMLVCFIDNGIHYHHDL

>AUF72985.1 AchiOBP38 odorant-binding protein 38 [Anoplophora chinensis]

MKTVFVFLCVIVGILAQDKPKLIAEEQMLEHIHDECQADPKTFADHDLLHNLAANIDNPQVGAHMLCESTKVG
LQKENGELDIETIKSKIGLSVADPNRVEFLVKECAVKKNTPEKTAINLFMCLDKNGVTYFHEF

>AUF72972.1 AchiOBP25 odorant-binding protein 25 [Anoplophora chinensis]

MRTCAIVVCIATLVVSIHCASEEQHERVKKIHSECQADPKTHADDELLKKYHNGEEVDKSIVGAHMLCMSTKFG
VIHEDGKINKSALKTALSRLISDETKLNEAIEKCAVEKDDPKDTALALGRCFRQQGGLTGHEHIHNRL
>AUF72950.1 AchiOBP3 odorant-binding protein 3 [Anoplophora chinensis]
MNNVVAFCALLVATVSAYNFEDPDFNILLSDDLEELSSGVASFSHPRSRRDDEAVNDKDKCHHRKRWGELCCAE
DVVAKMRDVEKDLKRECFKEVVGKDKHDKFDPFNCETMDQRKKQIVCVIECVGKKKDLLDTEGNPKEEEFRSF
LKESFSSESWLAALQDKVISTCLDEGKNATANRDASDSTSCNPAGIKIAHCLHREIQLNCPADQIKDEKSCARLQE
RLKRRDFFHPPPPPGGFDEPDN
>ATL75740.1 AglaOBP2 odorant-binding protein 2 [Anoplophora glabripennis]
MKAFAVVLCAVFAVALAQLPDSERIHLKQVHDSCQADPKTYADESRLKQLNKYIDDNVVGTHMLCMSRKAGL
QKANGDLDVAVIRQKIDLVTADKSKVDGLVKKCAVVDGNPKRTANLLWLCFVQNNIDYLHRL
>XP_018577241.1 AglaOBP70 general odorant-binding protein 70 [Anoplophora glabripennis]
MFPSVVCAVGSYLIFLTIIQAKEISSGRCDIPPSAPKKVEEIINQCQDEIKLAILSEALQAFNVNEHSRSRAKRATFSE
DEKRIAGCLLQCVYRKMDAVNEKGFPTVQGLVSLYTEGISQKDYILATVQAVNTCLIKSQKKYLITPQSIDENGKT
CDIAYDVFDCVSDEIGKYCGQRP
>ALR72513.1 CbowOBP25 odorant binding protein 25 [Colaphellus bowringi]
MYYALLPRSAVLAVIIVVVAAYNFEDTEFNQILANDLEDVYSFTYSHPRSRRDDKAVEEDKCHPPRRGRPLCCAEE
TMRKLHDDKKEIKRACFKEITGKEKPERPDRHHGPPFDPFSCEKIEQHRRDMMCIQQCVGEKLDYLDADGKPK
PEQFEKYVEGIFEKEDYLLPLKDKIVSVCLDEAKNATEKVSSDPCKSTGLVLEHCIFINTQLNCPEDQIKDKKMCSK
FQDRLRQGFDKRSSPSPEAEDE
>XP_044748578.1 CsepOBP19d general odorant-binding protein 19d-like [Coccinella septempunctata]
MFKFVVLLTCAFVAVNAVSEELKGKFLEKMTKIGGECAKEVGANEDDIAELMAHKLPSRHEGECMIFCFHKHLG
LMNEDGTLNKEGAMKAAEPLKADDPELHEKVVKIGKECAEEVAADDDKCKYATKLTECVVKKGKAMGLDSSVL
H
>ALR72502.1 CbowOBP14 odorant binding protein 14 [Colaphellus bowringi]
MSTTMKLAVFLFVVFSSVMEGYSAPSDSSNSTIKDYCIKEVEISEEKVNKFEKNPDDTPDEDIMCYTHCILVTLGV
VDDDGKIIIEKFTKIFDKYDMECVKKIPKILECTDLINLNKCAATDE
>AIX97067.1 DhelOBP21 odorant-binding protein 21 [Dastarcus helophoroides]
MKSFAFVLALTFVVAVYGLTEEQKEKIKAYHKDCSASSGVNQDLITKARKGEFVEDPKLMEHLFCFSKKAGFQNE
AGDIQTDVIKAKLGAEIKDSATVDQLIKKCAIKKATPQKTAFDTIKCYYESTPQHISLA
>AQY18970.1 GdauOBP6 odorant-binding protein 6 [Galeruca daurica]
MTEKQMNATKKLVRNSCTAKTKVAPDVVDAMHKGDFSNGQCYILCIMNTYKLIRADGSFDWEGGIATVNAN
APPHIAATAAASIKNCKDSMKNTSDKCLGSAEIAMCIYNDDPPNYFFP
>AQY18965.1 GdauOBP1 odorant-binding protein 1 [Galeruca daurica]
MYTLWMISVITMFAITVSASEFDDMRDRIMETKEYKECSQESGATYDDFMAFKNTTEVMCLFKCSLEKKGSLD
KDGNIDLDNIKERLSGNTHLDDTKKEMFLKCAESVGKIDKCDDLLEFRWCLVNITKKQ
>AQY18986.1 GdauOBP22 odorant-binding protein 22 [Galeruca daurica]
MYFHAFVFLFVAVISAEALSQLPGRFNKAHDKCQSDPASYVDEAILEKVRRGEKVTAPNLAKHTLCMNVESGVQ
DQNGEIIVENLRKFLERAGNTKEKVDEAISKCGTRTSAIAEEAAVALTNCVIKFRPERRHNR
>AQY18972.1 GdauOBP8 odorant-binding protein 8[Galeruca daurica]
MAKLLTLITIGVMCLSPSFAQKEDFIACLQESGLSDISEVKNLNTKDLSCFIKCTMTKTGEIDSEGNVNMDKINLN
LDKMKLTETIKDSLKTCFAGIEKIEKCEDAQKVTKCWSGVFNDTKWFDEQ
>AQY18988.1 GdauOBP24 odorant-binding protein 24 [Galeruca daurica]
MKSVIMFIIICFSFVLEIFAETRTAPNLEYQEARRKCQDDPKTTLDRETLKNAFRGDINTALFGGHAVCIYRHLGILD

EQANINKIKLRDSLDYIIVDQRQLDAAVKECSVKKNTPEETAVEIFKCTKRAMAPYLPHYEP
>AQY18979.1 GdauOBP15 odorant-binding protein 15 [Galeruca daurica]
MNKPIIFAVLCIGVASCFVPETEFGIKLLKLGKEAHEKCIEETGVTQAAIERAKQGKFDDDDIQIKDYNNCLWTFSK
AINKNFEINVELIKELLPAKIRDVQLKAIMDCHEEIKEGPILSLLEKTYLLSACVFNKNPENWIYF
>AQY18974.1 GdauOBP10 odorant-binding protein 10 [Galeruca daurica]
MSSMKYIFLSLCLFFIYENVQTLMTEKQIAATKKLIRNTCINKQGVAPEKVDGMYKGEFDFSDKNSMCYVHCVLT
TYKLIKKDNTFDWEEGISVMQLNAPPSIATPVIETIKNCKNAVKTTNHKCTAALEIAKCLYDDDPVHYFLP
>AQY18989.1 GdauOBP25 odorant-binding protein 25[Galeruca daurica]
MKTVFVFLCVLVAVLARGLPNLPENERIKLAEVHRSCQSNPKTFCDENKLKNLFNNINDPQVGTHMLCMAVKA
GLMRPNGDFDIPYLKQKVGLVVRDHSKVDGLIQKCTHKAENSGKTANLMWKCLVENDVQYYHQL
>XP_045479958.1 HaxyOBP19d general odorant-binding protein 19d-like [Harmonia axyridis]
MFKFLFLVACALVAVNAVSEQLKNEFIEKMTNIGGQCAKEVGANEEDIAELLAHKAPSRHEGECMIFCFHKHLG
LMNEDGTFSKEGGLKALEPVKADDPKLYEKLISIGKMCQEEVAKDDDKCKYATQLTVCGVKKGKEMGLDASMI
H
>QTE76124.1 HaxyOBP16 odorant binding protein 16 [Harmonia axyridis]
MNKVVLFGLVATALCAVAAYEFASDDFNEYLADELDYYSTMDVPIPKLRFRRDEEAVSDKCRYRRKRLCCAETSIE
ELHEKEKEIKRECFKQVLGKEKEHRMDPFKCENIEKHKRDMICVMQCVGQKNDVLDADGDVRDAEFADFVKQ
SFSKDPWFAEFQDTIIETCIDEAKNATEIRDTEDKSACNPAGIKLAHCLFVQTQLNCPESEIKDTKSCSKLKERIKAK
LSDGVPPPPPFLHEE
>AEA76516.1 HparOBP2 odorant-binding protein 2 [Holotrichia parallela]
MKYFVLFAALCTYVLGATLQEHHQVVMEQIINAGAECAEKLGATPEDVDKLMKEQIPDNKAGLCVVACVNKKF
GLQNDDGTVNKGSTLVNIEKVKDIDEDMYKKMSSVWSTCNENTGNDSDECNTGINLLKCMKEEGGKLGLTKE
SMGF
>AKI84378.1 HparOBP20 OBP20 [Holotrichia parallela]
MKFFVLLSFVAFANCMDQDFLTKATARMSQYVEECAESTGATKDDLAEVMEIRIPSSKEGKCFLACYHKKYGIQ
DKDGKLDKEASIEAMSDLKIADPELYDKAVKLFDTCINQVPNQDCECNTAAIFMYCFNINGGMMGLKPGMVP
M
>AKI84360.1 HparOBP49 OBP49 [Holotrichia parallela]
MKISLFCFALCVAIVNASIEKYIDQLTTEIATAKTECAKQVGASVDDVVEVFQGKTPTSKEGKCIISCVLKLFGGQDS
NGKINKHAAIGKIEELKPIDTDVYEKFSSVWKSCSEKTSDDNDGCDSAAKLMICLAQEVDKHGISKKLIGL
>ADF87391.1 HparOBP49 pheromone binding protein 1, partial [Holotrichia parallela]
MFKNTALLILGTFLPTVLCLSEEMEALAKQLHDDCVAQTSVDGAHITTVKDQKGFPDDEKFKCYLKCLMTETAIV
GDDGVVDVEAAVGVIPDEFKDIAEPIMRKCGVIPGANPCDNVYQTHKCYYEMDPKSYMIV
>HparOBP14 OBP14 [Holotrichia parallela]
MKFVIVLLLSALAFINAYDFEDHAYNELLVEQYSLAHPRLRRAAENNCIDSRSCCYNDAVPQYHANETEECSKELG
FDRSQIRGPLTDAQINQIKCLAECISKKKGHFDADGNLLKDELLKDIRKPLEQVAWLKPKVEDIFNKCLDGLPTKV
PENQCNDIGMTIGHCIWKEIQLECPKDRQANLEKCEALQVYLKENKAFPPPPPIRC
>XP_023018007.1 LdecOBP70-X2 general odorant-binding protein 70 isoform X2 [Leptinotarsa decemlineata]
MIASELILEFLLILAVKLYNGCDIQALAAINMNEHTHSRAKRAAFTDDEKRIAGCLLQCVYRKMNAVNGEGFPTV
DGLVSLYTEGISQKDYILATMQAVNHCVNHAQKKHLITPQSISEHGKTCDIAYDVFDCVSDDIGKYCGQTP
>AIX97039.1 MaltOBP24 odorant-binding protein 24 [Monochamus alternatus]
MSQVREGEKDLKRECFKEVVGKDKHEKFDPFNCETMDQRKKQISCVIQCVGQKKDLLDSEGNPKEEEFRAFVK
ERFASESWLAALQDKVISACLDEAKNATANHDASDSASCNPAGIKIAHCLHREIQLNCPADQIKDEKSCARLQER

LKRRDFFHPPPPPGAFDEPDN
>AIX97023.1 MaltOBP8 odorant-binding protein 8 [Monochamus alternatus]
MKILVTFVCVSIACVLVYGAPSIRDVHNECQADPATHIEDAVFKAIHDGESFDKVKVGAHMLCMATKSGIINQD
GTINKSAVKELLAVDISDETKLVEVTNKCAEEGPTPSETALKLSKCITENTKRGRHGHGHHHGHHHEHHHDHSV
H
>AHA39267.1 MaltOBP2 odorant-binding protein 2 [Monochamus alternatus]
MKAVLVLCVVVAGALAQLPDSERIHLKQVHDSCQADRATYADESRLKQLNKYIDDAVVGNHMLCMSKKAGLQ
KGNGDLDIGVIKQKIALVTADKSKVDGLVKKCAVVNGNPQKTANLLWLCFVQNNIDYLHRL
>AIX97019.1 MaltOBP25 odorant-binding protein 25 [Monochamus alternatus]
MFLRVVCVVGSLLVFLIVVQAKDAPSGRCDIPPSAPKKVEDIINQCQDEIKLAILSEALQAFNVNEHTRSRAKRAT
FSEDEKRIAGCLLQCVYRKMNAVNEKGFPTVEGLVSLYTQGISQKDYILATIQAVNTCLFKSQRKYLITPQSIDENG
KTCDIAYDVFDCVSDEIGKYCGQTP
>AIX97024.1 MaltOBP9 odorant-binding protein 9 [Monochamus alternatus]
MRTCAIVVCFATLVVSIQGLTDEEKGRIKTVHSECQADPKTQIDDELLKKYYKGEHVDKSVIGPHMLCMATKFGT
VNAEGKLDKSKLRQGLSRLISDETKLNEAVDKCSVDKDDAQDTALAVERCVREQAGIAGHVHHHH
>AIX97020.1 MaltOBP5 odorant-binding protein 5 [Monochamus alternatus]
MMKLGFILSCVIFGALAASLSEEEKKLQEIHDKCQADPATYVDHELLHHLAENIDNPKVGAHMLCESKAVGLQK
QNGELDLNVIKQKISLTVSDKVKVERLVKECAVKKETPEKTAVNLFMCLDKDGVTYFHEF
>APC94280.1 PaenOBP26 odorant-binding protein 26 [Pyrrhalta aenescens]
MKILALICFLGIFLSVYGDSHFLFMAKYAKYYSDCDSEPATALNRDDFKNAMSGKPFNAEQAAYFFCLNTKFGVQ
NEQGDYVREPLKKFFKHIVVNQDNIEKIVEICSTRPGGSSAKDAALSLFNCAKKIDNLHFPFQHFTIEA
>APC94281.1 PaenOBP5 odorant-binding protein 5 [Pyrrhalta aenescens]
MKTLALVCFSAIVLPVYADSYFSFYANYITYYMLCDNDAATSLNRDDLRNALRDKPFNAKQLEAYLFCLNKHFGV
QNEQGDYVPEKLKEFFKHVVVNQDNLEKIVHSCSTRPEGSSAKDAALSLLNCAKKFDTLNIPFLNETSES
>APC94279.1 PaenOBP4 odorant-binding protein 4, partial [Pyrrhalta aenescens]
RGLVQILKRLTMHFSVFVLFFVFAVASAQINPEQRESFKKIHDKCQSDPATHVDDAVFDKIRRGEKVTEPNLAKHT
LCMNVGSGIQNENGDINIEKLRTLLANGPGNKEKVDEIIGKCGTRSSTNAVEAAEALSNCLRQYRPDRPEGDHH
DHHDHDHHHHH
>APC94285.1 PaenOBP29 odorant-binding protein 29 [Pyrrhalta aenescens]
MIKFSILVTIAAICVCTSMAQKGSFVACLQESGLPEDFSKFGSPDSKEVNCFVKCMMTKMGEIDEEGNVLMDKI
EENLANIKIPDTAKDNLRKCLSAIDKIGKCEDSKKVTECWTSLFK
>APC94277.1 PaenOBP2 odorant-binding protein 2 [Pyrrhalta aenescens]
MKTIFVLTCIVAVALAKNLPNLPENERVKLAQVHQSCQSDPKTYCDESKLRNLVNNIDDPVVGTHMMCMAVK
AGLMKSNGDFDLGTMKNKISLVINDQSKVDGLVKKCSHKTDNLGKTANLMWVCFVQNDIQYYHKL
>APC94283.1 PaenOBP7 odorant-binding protein 7, partial [Pyrrhalta aenescens]
RVLTLIIPNTNKFTMKTILVFSLLLAVTLAGNRREEIKEAVTACGGENDGPNELGKQILKENADQAKLGAITFCVHK
KLGIQNDHGDIDVDVIRKDLNALIKDESEVNKIVEECGKRNGDTPEAAAFNFFKCYQKHVTK
>APC94274.1 PaenOBP8 odorant-binding protein 8 [Pyrrhalta aenescens]
MKVIVFCLMLFTFALANETKELIEGSLKSCGAEIDGPNAIRTLVVQENADEKKLGAILFCANKKIGLQYADGNINL
DVLERLIEAGNNDEETAKKYMDCGRLKGENGEEKAFNFLKCHETI
>APC94266.1 PaenOBP23 odorant-binding protein 23 [Pyrrhalta aenescens]
MKFVLLAVLCIGSISCFKESEHNEKFQKASKEVHRICMEETGITQEAIDNVKKGNFDDDSKTKKYNNCLWTRIKV
MNEKFELDEKLLKDLLPERIRDVQLKFIIECLEETRAAAADLESVEKTYLLTKCIYNKKPENWFYI
>APC94265.1 PaenOBP31 odorant-binding protein 31 [Pyrrhalta aenescens]

MKAAIILALCLAIAHCLVDVEELGEEFVEKAKHLHDVCVAQTQVPNSVINLYKAGIFPVQNNLLKKYLTCLWLESK
GANRNLEFNDEVIKNFFPASIAAHLVPIYVGCAKLVKQSAPMSTPIDDKFYLISNCIYFKDPENWVFF
>APC94264.1 PaenOBP22 odorant-binding protein 22 [Pyrrhalta aenescens]
MKTALIFTLCLAAAQCIDLKELGDEFVQKTKKLHDLCVAKINVTDSEIEQFKAGVYSVQNKLFKEYVACLWLESKA
VDENLNVNEEVVKNFLPPSKTVHLAPVYVACIKLVKKTVPANAPVEEKFFLISNCVYFRNPEDWIFF
>APC94273.1 PaenOBP10 odorant-binding protein 10, partial [Pyrrhalta aenescens]
PGVLQLSRIKKHLTTMMSYSIVMCVVLALSTALPSPDDSSQKRMQESHEECLKENGIEEIALSKVLEAKVSEEALE
NYSYCYGKKYGYIGKDNKIDRDIAKQVFEAMIPGSSEEFMKTCIPDGEVTKKIALEINRCIDRKWKELKKN
>APC94276.1 PaenOBP1 odorant-binding protein 1 [Pyrrhalta aenescens]
MKTVFVFLCVLVAALAHGLPNLPENERIKLAQVHRSCQSNPKTFCDENKLRNLSNYINDIEVGTHMLCMAVKA
GLMTPNGDFDIPSIKRKVALVTRDHSNVDGLVQKCTRKAEKPGKTANLMWMCFFQNDIQYYHQL
>APC94282.1 PaenOBP6 odorant-binding protein 6 [Pyrrhalta aenescens]
MKIFFIVSLLIAATLANEYIEKLETAAKECGIGGVGELRALEAKGEKTTIGPTLFCMNKKIGIQDSNGIVHAEGVAN
HIRKTHAEKDESEQNKIIENCDKPTGDSGADKAYNLFLCVIKNLH
>XP_031344875.1 PpyrOBPP70 general odorant-binding protein 70 [Photinus pyralis]
MYMLRLFSIIVVLTIVGVHAKDLKCQVPIAAPKNIEKVVNQCQEEIKTALLSEAFHFLTDNQSVNSSRRKRETFTEE
ERRIAGCLLQCVYRKMKAVNSNGFPTAEGLIALYTEGAQDQEYLLASHQAVSQCLVDAQKKHLPTPHSITIKGKT
CDIAFDVFDCVSDRIGEYCGQSL
>APC94202.1 PmacOBP4 odorant-binding protein 4 [Pyrrhalta maculicollis]
MHCFHKYLRIFVDEIRIVIKARACIIFVLVQILKRFTMHFSVFVLFFVFAVASAEITPEQQAEQFKKIHDKCQSDPAT
HVDDAVFDKLRRGEKVTEPNLAKHTLCMNVGSGIQSENGDINIEKLRTILERGPANKAKVDEIIGKCGTRSSTNA
EEAAEALANCVRQYRPERPEGDHHGPHDHHHHH
>APC94203.1 PmacOBP31 odorant-binding protein 31 [Pyrrhalta maculicollis]
MFCGSIIFISVCAIIVLGAESKQTNYTNKCEIPASAPRKVEEFINQCQDEIKLAILSEALQTLNANENAHSRAKRAAF
TDDEKRIAGCLLQCVYRKMKAVNEHGFPTTEGLVSLYTNGIEQKEYIRATIESVNTCMKMAEQKYLIKPDSIDEN
GKTCDIAYDVFDCISEEIGKYCGQTL
>APC94190.1 PmacOBP29 odorant-binding protein 29, partial [Pyrrhalta maculicollis]
KALLKASSAPVTSKKPEYNTHITMMSFSIVLCVVLALSSALPRPDDASEKKMQESHEECLKESGIEEIDLRKVLETE
APEEALENYSYCFAKKFGFIGEDNKIDRDIAKQQLEAMVPGSSEEFLKECIPEEEVTKKIALEITRCIDSKYKELKKD
>APC94189.1 PmacOBP10 odorant-binding protein 10, partial [Pyrrhalta maculicollis]
IKKHLATMMSYSIVMCVILALTTALPSPNNSIKERMQESHEECLKENGIEEIVLSKVLEAKVSEEALENYSYCYGKK
YGYIGKDNKIDRDIAKQEFESMVPGASEEFMKTCIPDGEVTKKIALEINRCIDRKWKELKKN
>APC94199.1 PmacOBP1 odorant-binding protein 1 [Pyrrhalta maculicollis]
MKTVFVFLCVLVAALAHGLPNLPENERIKLAQVHRSCQSNPKTFCDENKLKNLSNYINDIEAGTHMLCMAVKA
GLMSPNGDFDIPSIKRKVALVTRDYSKVDGLVQKCTRKAEKPGKTANLMWMCFFQNDIQYYHQL
>APC94193.1 PmacOBP5 odorant-binding protein 5 [Pyrrhalta maculicollis]
MKILSLICFFAVFVSVYADSYFVFCAKYITYYIMCGNDAATSLNRDDLKNALKDKPFNAKQLEGYLFCLNKHIGVQ
NEQGDYVPEQLKEFFKHVVVNQDNLEKIVHSCSTRPIGSSAKDAALSLLNCAKKLDTLNFPFLNSTLQTDISSI
>APC94194.1 PmacOBP6 odorant-binding protein 6, partial [Pyrrhalta maculicollis]
RELSDISIMKIFFIVSLLIAATLANEYIEKLETAAKACGLGGLGELRALEAKGEKTTVGSTLFCINKEIGIQDSNGIVKA
EEVANHIRKTHQDKDVSVQNKIIENCDKPTGDSGVDKAYNLFLCIIKNLH
>APC94180.1 PmacOBP23 odorant-binding protein 23 [Pyrrhalta maculicollis]
MKFILLAAVCIGGVSCIILESEFNEKLQKISKEAHRICVKETGITQEAIDHVKKGNFYDDLKTKKYNNCLWRNTQV
MGENFKLDEKLLMELVPEKARDLLFKGISECFEEARAAPADLDFIEKTYSLSKCIHDKSPENWFYF

>APC94197.1 PmacOBP27 odorant-binding protein 27 [Pyrrhalta maculicollis]
MKSLLVCLLIITLAMTDETKDLIEEALISCGAEIEGPNNIGTLVVQENADETKLGTILFCANKKIGLQHDNGEINIDV
LENLMETGNRDEATAKKILDCGRSKGDNGAAKAFNFLKCHELAVRP
>APC94179.1 PmacOBP33 odorant-binding protein 33, partial [Pyrrhalta maculicollis]
MKTVIILALFFVAANCAINPAEFSPKLIKLTQELHDTCKSQSNVKDEYIQAHKQGNFAVNDITIKKYIACLWSVSKA
LDEHLVGDYDFLYSVIPPRVSSYLVPLYLDCFRQVRLTVPADAPFEEKTFLINTCIYYRNP
>APC94177.1 PmacOBP22 odorant-binding protein 22 [Pyrrhalta maculicollis]
MKTALIFILCLAAAHCIDLKEFGDEFVQKTKKLHDLCVAKINVTDSEIEQFKEGVYSVQNKLFKEYIACLWLESKAV
DENLNVHEDVVKNFLPPSKTGHLAPAYVACIKLVKKTVPANTPVEEKFFLISNCVYFRNPEDWIFF
>APC94187.1 PmacOBP28 odorant-binding protein 28 [Pyrrhalta maculicollis]
MKLFISFLIVAIVCCANAELTAEQKEKVKGYHKECIDVTGANPDLVKQAREGKFADDEKLKNHILCVSKKIGFQND
AGDIQTDVLKHKLGAALGDQAVADQLISTCAQPKANPAETAFQTLKCYYEKTPTHVSFI
>APC94196.1 PmacOBP26 odorant-binding protein 26, partial [Pyrrhalta maculicollis]
EETPGLNNNSTSTMKTVLIISMLLAVSLAGKRREEIKEAVRSCGGDAGGSDDLRKHVTDENVDQAALGAKIFCV
NKKIGLQNDHGNIDESVFRKDLKAVVGDEAKVNEIVAECGKRKGDTPEAAAFNFLKCYEKNVPK
>APC94195.1 PmacOBP7 odorant-binding protein 7, partial [Pyrrhalta maculicollis]
AASNKENTSKFTMKTILVFSLLLAATLAGNRREEIKEAVTACGGENDGPNELGKLILNENADQAKLGAITFCVNKK
LGVQNDQGDLDVDVIRKDLNTFIKDESEVNKIVEECGKRKGDTSEAAAFNFFKCYQKHITK
>APC94198.1 PmacOBP8 odorant-binding protein 8 [Pyrrhalta maculicollis]
MKVIVFCLMLSTFALANETKELIEGSLKSCGAEIDGPNAIRTLVVQENADEKKLGAILFCANKKIGLQYADGNINL
DVLERLIEAGNNDEETAKKYMDCGRLKGETGEEKAFSFLKCHETI
>XP_030765038.1 SoryOBP70 general odorant-binding protein 70 [Sitophilus oryzae]
MTGMGRILIFVFFIYGCQAVQDIAKKCDIPATAPKKIEEVINTCQDEIKIAILSEALEVLSVNEEKARVKRSTFNPDE
KRLAGCLLQCVYRKLDAVNENGFPTEEGLVTLYTEGVTQKEYVTATKQAVQTCLTQVQKKLLVTPLSLEESKVCDV
AYDVFDCVSEEVAKYCGQTP
>QCT83274.1 SzeOBP20 odorant binding protein 20, partial [Sitophilus zeamais]
MTGMGRILIFVFFIYGCQAVQDIAKKCDIPATAPKKIEEVINTCQDEIKIAILSEALEVLSVNEEKARVKRSTFNPDE
KRLAGCLLQCVYRKLDAVNENGFPTEEGLVALYTEGVTQKEYVTATKQAVQTCLTQVQKKLLVTPLSLEESKVCDV
AYDVFDCVSEEVAKYCGQTP
>QCT83286.1 SzeaOBP32 odorant binding protein 32, partial [Sitophilus zeamais]
MNTLVALCVVVSAAVSAYDFQDVEFNQLLASDLEDGFDSLDSLPLNSRVRRAQEAATQSPNEDDKCKKRHRRP
KACCADELLDQIHEKDKELVRSCFREVLGVDKHEHHRRGDPFSCEQAEKRRNEMTCVFQCVGQKKGVVGEDG
KPKPEEITKTLKENFATESWFEPSIEKVVSTCLKEAENATATQVKPASADVKICNPSGITLKHCIFREIQLSCPADQIK
DQKACERFQERIKKGIDFFEPPPPPFDGPRDE
>SvelOBP4
MHSCQNSYFVVVLFVLNVLISVNCQSDQAGGPPGKHPFHAASKEDFQKCLDETGLTIDDLKSLHHDPSKEPTEK
HLCFMKCTATSGGLISKDGTLMTDVIKQNAPPYLDETALNTAIKCLEGVGKISTCSDMSKVFKCFPKPPNRPQ
>SvelOBP15
MVTKKSKMCQVYCCIALVFCATSVLAKVDESIFTDDLKKLMDNLHETCVQKTRVSEALIDKVKEADFVDDDRLK
CYVKCVMTESGTMDESGEIDYQALMEVVPDKYKISIKKDFENCSRKADGIDDLCEKAYTILQCVQEIDPDNYYFV
>EFA07544.1 TcasOBPC01 odorant binding protein (subfamily minus-C) C01 [Tribolium castaneum]
MKTIICFVFVLAGAWALTKEQIDKLEPISKECRELNGISEDTILKVRRGEAVNEPKLKNHVLCVSKKTGLASETGET
NVEVLRTKLRKVSENDDEVNSIIQKCVVKKSTPEETAFEIFVCLRKVKPNFSPAN

>EFA07546.1 TcasOBPC03 odorant binding protein C03 [Tribolium castaneum]
MKFLVCLLFVIVAANALTKEQKEKLDKISKECKNQSGVSQELIDKARTGELINDPKLKAQIYCVSKKAGLATEAGEI
NMDNLKTKLKKVAANDDEVNKIIQKCVVKKPTPEETAFEVYKCLHANKPNFSVVD
>EFA12066.1 TcasOBP15 odorant binding protein 15 [Tribolium castaneum]
MNCFVIFALSLSATVFGQSLSEDEMRENARKLMTSCKDKVGASDADVEALKMHQMPESREGFCMLECVFDSA
KIMQDGKFSKSGMIEGFKPLIGDDKAKLESLEKLSATCESELGDGEDKCETAKRLVECVIKNGKTHGF
EVPPPRE
>XP_044268412.1 TmadOBP70 general odorant-binding protein 70 [Tribolium madens]
MCTKVCILILSIIIIVNAEESKENKCNIPPTAPKKIEDVINKCQDEIKLAILTEALEALSINEHAKSRAKRDTFSDDEKRI
AGCLLQCVYRKMKAVNEKGFPTVEGLVALYSEGVTQKEYIIATLQAVNVCLNKAQKKHLTKPQSLEEHGKTCDIA
YDVFDCVSERIGEYCGQTP
>EFA07430.1 TcasOBPC04 odorant binding protein (subfamily minus-C) C04 [Tribolium castaneum]
MKAFIVLVAVAVCAQALTDEQKEKIKNYHKECSAVSGVSQDVITKARKGEFIEDPKFKEHLFCFSKKAGFQNEAG
DFQEEVIRKKLNAELNDLDATNKLIAKCAVKKDSPQQTAFETIKCYYENTPTHVSLA
>EFA07518.1 TcasOBPC14 odorant binding protein C14 [Tribolium castaneum]
MNIYTCLVLVVIAASAQHLTEEQKNNWRKWSNECKVLIGVSQEAINKIRNNEFDSVDDKIKKHGLCFAKKASLA
DSSGNIIINQIKIKLKRVIEDDEEVDRIVTKCTIRKNTPEETTFETFRCLRENSSKFVPV
>EFA07543.1 TcasOBPC05 odorant binding protein C05 [Tribolium castaneum]
MKFLVVISTVLMANIVQGLTDEQKSKLEEYSKECLKESKVDESVLKEAEKGVYLDDPKLMNHVYCLVKKINSQKD
KGELEVTQIKEKLMMQINDEKEVDKLIQLCLVQEKSARYSLGKCEVSS
>EFA02860.1 TcasOBP18 odorant binding protein 18 [Tribolium castaneum]
MKLFILAGILFTGVCAVDQEFVEKFLQKMEKIGEECAEETHATSDDIADLIEQRDPKTHEGKCLIFCYHKKFNTMK
EDGSLDKVGSVLALEEVRDADFELYKNILTIFVTCGDKAKIYDDPCETATALTMCGRDEAKALGLQDAIFG
>EFA02861.1 TcasOBP17 odorant binding protein 17 [Tribolium castaneum]
MKSTWFFLLLACSLTCALDQEFVDEFLEKMQEFGAQCAEETDATSDDIAELIARKLPPSTHEGKCMIFCMQKKF
NMMKENGGIDRAGAIAALKPLQKADPELHQKVLKIFVTCGMRVKPSPDPCDTATELALCGKKEAEAIGLEDALL
T
>AXO78390.1 XquaOBP12 odorant binding protein 12 [Xylotrechus quadripes]
MKSVIVFLCAIVGILAQNRLIVEEQMLHHIHDACQANPATYADHDLLHDLSANLDNPQVGAHMLCESKGVGLQ
KENGQLDPDTIRDKISLTIGDAAQVDRLVKECAIPKNTPEKTAIHLFMCLDRNGVTYFHEF
>AXO78393.1 XquaOBP15 odorant binding protein 15 [Xylotrechus quadripes]
MKPVLVFLCAIVVGITGQNRRIVEEQNLHHIHDACQADPATNADHELLHNLAANIDNPQVGAHMLCESKGVGL
QKPNGELDPETIRSKLTVFIDDPAKVDKLIKECAIQKNTPEKTAIYLFMCMDKEGVTYFHEF
>AXO78394.1 XquaOBP16 odorant binding protein 16 [Xylotrechus quadripes]
MKGLVFIVCVTISVTLASGLTEEQKEKVKIVHKECQDDPATHADENKLSDSYKGVEVTGLGAHFLCMSKKLGMQ
KDNGNVDREAIKRKLSDVITVPEKLQEAVDKCAVQKATPEDTAEHVLKCFHDYAGHVLHAHDHHH
