# Supplementary material for: Antennal Transcriptome Evaluation and Analysis for Odorant-Binding Proteins, Chemosensory Proteins, and Suitable Reference Genes in the Leaf Beetle Pest Diorhabda rybakowi Weise (Coleoptera: Chrysomelidae)
Source: Insects. 2024 Apr 7;15(4):251. doi: 10.3390/insects15040251 (PMC11050234; doi:10.3390/insects15040251)
Supplement: Supplementary file 1 [file insects-15-00251-s001.zip › Appendix B.pdf]

Phylogenetic tree of DrybCSPs

>OR797794 DrybCSP1 chemosensory protein 1, complete cds [Diorhabda rybakowi Weise]

ATGAAATTTTCTCTTCTCGTTTGTGTTGTATTAGTGATAGTAGTGGTTTCGGCCAGACCCGAAGACACATATAC
AACAAAATATGATAATATTGATATTGACGAAATTATTCACAACGATAGATTATTGAGGAGTTACGTGGATTGTC
TGATAGGAACTAAGCCTTGTTCCAAAGATGGAGAGGAGCTGAAAAAGGTTTTCAGGGAAGCGTTGGAAAC
TAAATGTTCTAAATGTAGTGAACCACAAAAAGAAGCTTCGAAGAAAATAGGCAAATACCTGATAGAGAACAA
GAGATCATGGTTTGATGAGCTTGTAGAAGTTTTCGATCCAGACCACTCCAAATTGGAACTTTATAGAGAAAA
TCTTAAAGCTGAAGGAATTGAATTATAA

>DrybCSP1

MKFSLLVCVVLVIVVVSARPEDTYTTKYDNIDIDEIIHNDRLLRSYVDCLIGTKPCSKDGEELKKVFREALETKCSKC
SEPQKEASKKIGKYLIENKRSWFDELVEVFDPDHSKLELYRENLKAEGIEL

>OR797795 DrybCSP2 chemosensory protein 2, complete cds [Diorhabda rybakowi Weise]

ATGAAACTAGCAATTGTAGTTGTTTGTGTTTCACTAGTGATAGTGGCAGTTTCTGCAGCACCATCTGATGAAA
AATATACCTCAAAGTATGATAATGTTAATTTAGATGACATTATCAAAAGTGATAGATTACTAAGGAATTATATTG
ATTGTCTTCTGGGAACCAAGAAATGTACTAAAGATGGCGAAGAATTGAAAAGGGTTCTACCGGACGCATTG
AAAACCAAATGTGCTAAATGTACTGACACTCAAAAAGAAGGAGCTAAAAAGATTTTAAGACATTTATTGAAA
AATAAACGACAATGGTTCGACGAACTTGAAGCTTATTACGATTCCGACCACGTTTATCTGAAAAGCTACGAA
AAAGAGCTTAAAGAAGAAGGAATAGAAGTGTAA

>DrybCSP2

MKLAIVVVCVSLVIVAVSAAPSDEKYTSKYDNVNLDDIIKSDRLLRNYIDCLLGTKKCTKDGEELKRVLPDALKTKC
AKCTDTQKEGAKKILRHLLKNKRQWFDELEAYYDSDHVYLKSYEKELKEEGIEV

>OR797796 DrybCSP3 chemosensory protein 3, complete cds [Diorhabda rybakowi Weise]

ATGAATCGTTGGTGCATATCGGTCGTACTCATGGTATTGATTGTGGTAGCAGCAGTGCACTGTGCTCCGAAGA
GTTATGATGAGAATTTGAAGATTATTAAGAAAATAGATATCGATCAAGTGTTGAACAATGATAGAATTATCAGA
AATTACGTCGATTGTGTCTTAGGAAAGAAACGTTGTACTAATGAAGGAAATGCTTTGAAAGAAAGCTGGAA
AGACGGTTTAGACAAAGGATGTGATAATTGCGATGAAGAAGACAAGAGGAAAGTGAAAAAAATCTTAAAA
CACATATACACCAAACACCGAGATTTATACGATGAATTGGCTGCCCACCTTGACAAAGATGGTAAATACCGTG
AAAAATACCAAGCTCAAATCGATGAAATTCTCAAGGATCAAACTCTATGA

>DrybCSP3

MNRWCISVVLMVLIVVAAVHCAPKSYDENLKIIKKIDIDQVLNNDRIIRNYVDCVLGKKRCTNEGNALKESWKD
GLDKGCDNCDEEDKRKVKKILKHIYTKHRDLYDELAAHLDKDGKYREKYQAQIDEILKDQTL

>OR797797 DrybCSP4 chemosensory protein 4, complete cds [Diorhabda rybakowi Weise]

ATGGGGTTAATACGATTATTTGTTTTATTGGCGTTGGTATCTTGTAGTTTAGCTCAAACTTACAATACTAGATAT
GATAACATCGACATTGACCGAATTTTAGCTAGTAAAAGATTGTTAGATAATTATATACAGTGTCTTTTAGAAGA
GAACGTTAAAAGGTGTTCACCAGAAGGACGGGAATTTAAAAAATACATACCTGAAGCAATTCAAACCAACT
GTGCTAAATGTTCAGATTCCCAAAAAAGAATTGTGAAAAAAACTGCCAAACATATAATAACCAATAGACCAC
AAGATTGGGAGAAAATTAAACAGAAGTTTGATCCTCAAGGAACATATCATCAAAGTTTCAATAATTTCTTAAA
TAGTCCATAA

>DrybCSP4

MGLIRLFVLLALVSCSLAQTYNTRYDNIDIDRILASKRLLDNYIQCLLEENVKRCSPEGREFKKYIPEAIQTNCAKCS
DSQKRIVKKTAKHIITNRPQDWEKIKQKFDPQGTYHQSFNNFLNSP

>OR797802 DrybCSP5 chemosensory protein 5, complete cds [Diorhabda rybakowi Weise]

ATGCAGATATTTCAGTTTGGTGTAATATTTTTGATCTTCCTGGTATCAATAACTACCGTAGCAGCGGCTGTTAC
AGAAAAATCTACGACTCTAAGACCTTCCATCTCAGATGAAGCTTTAGAAGCCTCCCTAAAAGACAAAAGATA

TCTACATAGGCAACTGAAATGTGCTATAGGAGAAGCCCCGTGTGATGCAGTAGGTCGTAGATTGAAAAGTTT
GGCTCCGTTGGTTTTGCAAGGTTCATGTTCTCAATGTACACCTCAGGAGCAACAACAAATTAGGAAAGTTTT
GGCATATATGCAAGTTAATTTTCCGAAAGAATGGAATAAGATATTGAAACAATATTCCGGATAA

>DrybCSP5

MQIFQFGVIFLIFLVSITTVAAAVTEKSTTLRPSISDEALEASLKDKRYLHRQLKCAIGEAPCDAVGRRLKSLAPLVL
QGSCSQCTPQEQQQIRKVLAYMQVNFPKEWNKILKQYSG

>OR797798 DrybCSP6 chemosensory protein 6, complete cds [Diorhabda rybakowi Weise]

ATGGTACCGTTAATCTGCATACTAGTTGCAGGGTTAAGTGGGTTAGTAGATTCGGCTCCAGTTTCTGATCAAA
ATCAACAATACTACACCACGAAATATGATCATGTCGATATAGAAATGATATTAAAGAATAGGAGATTGATTTTTT
ATTACACGGCGTGTATGCTCAATAAAGGACCATGTTCACCTGAAGGACTAGAATTTAAAAAACTTATACCAGA
TGCCATACGAACTAATTGCAAAAGATGTACCGACAAACAAAAAGTTACGACTGTTCGAGCAATACAAGGTTT
GATGAAAGAATACGAAAAAATATGGGAACAACTCAAAACCGAATGGGATCCCGACGATATATATGTGAAAAA
ATTCCTCGAAACGCACGGTAACGTTCCCAATCCGAATATAATTTTAAACAGATTCGATGGTGGTGAAGAATCT
GACGATACGTCCACAGAATCGTCTTCTACTACGTCTACTAAATCCCCTTACAATACTCCTCCAGTTATATTTTCA
GGTCCGGTACCAACTTTAAACCCAGAAACTATCCCTATAGCTAATGCAATTGGACAAAGTATAAAAGCTACGA
TTTATGGAATTTCTCAATTCTGTCATACGTTTAAAGTAAGTCTCATTGTTCTATTGCATCAAATTACATCAACTGT
GACTGAGGCCTCTGTCAAATTCTTCCTAAGACCTTAG

>DrybCSP6

MVPLICILVAGLSGLVDSAPVSDQNQQYYTTKYDHVDIEMILKNRRLIFYYTACMLNKGPCSPEGLEFKKLIPDAI
RTNCKRCTDKQKVTTVRAIQGLMKEYEKIWEQLKTEWDPDDIYVKKFLETHGNVPNPNIILNRFDGGEESDDT
STESSSTTSTKSPYNTPPVIFSGPVPTLNPETIPIANAIGQSIKATIYGISQFCHTFKVSLIVLLHQITSTVTEASVKFFL
RP

Phylogenetic tree of Coleopteran CSP sequences

>AXG21598.1 AmalCSP5 chemosensory protein 5 [Agrilus mali]

MGNRNWIFLSIFLTVLVTTLAQRHIIGRLQSVNLEQVLNNDRLFNNFYKCLADEGKCSNDAKSIKNLLPEVVKTK
CGQCTADERSQIKKSLNLVRKNKPEQFNSLAAKYDPEGVWKELVR

>ALR72526.1 CbowCSP12 chemosensory protein 12 [Colaphellus bowringi]

MKEGKARKLSQCYSFHGHEQCKYISLKMFLPFVVLSCLITLSISAVPEKSRYTTKYDNINLEEIIHNDRLLKNYVDCL
LDKGRCTPDGLELKKNMPDAIETDCSKCSDKQKEGSEIMMRYLIDNKPEYWNPLQEKYDPSGSYKKRYLDAKK
TEVSVEPIVKS

>ALR72519.1 CbowCSP5 chemosensory protein 5 [Colaphellus bowringi]

MKASALFFVAFFAVVATKPAEKYTTKYDNVDLDTILKSDRLLKNYVNCLLDKGNCTPDGAELKKVLPDALQTDCS
KCSDVQKRGSKKIIRYLIDNKAEWYKELEAKYDKNGVYKKKYDKELEDAKV

>AXF53965.1 DarmCSP2 chemosensory protein 2 [Dendroctonus armandi]

MKFCVVLVLVLQISICLCQTYSSRFDNINIDEILSNKRVLNNYVRCVLDEGPCTAEGRELRTHIPEALRTNCAKCTP
SQQKFVRKGASFLIKNDPDQWKRITKKFDPEGKFASQFHQFLNA

>QPZ89236.1 DadjCSP2 chemosensory protein 2 [Dendroctonus adjunctus]

MKFCGVLVLLLQIAVCLCQTYTSRFDTINIDEILSNKRVLNNYVRCVLDEGPCTAEGRELRTHIPEALRTNCAKCTP
SQQKFVRKGANFLIKNDPNQWSRITKKFDPEGKFAAQFHQFLNA

>AIX97072.1 DhelCSP4 chemosensory protein 4 [Dastarcus helophoroides]

MKFDIPLILIVTVASVKCVPVEYYATKYDHLDIERILNNRRMVNYYASCLLNKGPCPPQGTEFKRILPEALETNCAR
CTEKQKFVTYHTIKRLKKEYPKIWVQLEAQWDPNHTFIPLFEASFIASSTSPSTTSTTVVPDLGNRFGENKNETET
FPYPAEAVASGATLLPNLQQTTAFSSSIQSTHSTNTQHTTPPYLSYSTTKRYNVKQQQNTVSYGFNIVGDVIKNIG
IFSVRVAETGKQLANVLISSLHI

>ARM20140.1 GdauCSP4 chemosensory protein4 [Galeruca daurica]
MGLIRLIFLFAVVSCSLAQTYNTRYDNIDIDRILGSKRLLDNYLQCLLDENVKRCSPEGREFKRYIPEAISTNCAKCS
DSQKRIVKKTAKYIITNRPQDWEKIKQRFDPQGKYHQSFNDFLNSP
>ARM20141.1 GdauCSP5 chemosensory protein 5 [Galeruca daurica]
MFSLVVVLCLAGLSSAAVTEKAKYTTKYDNVNLEEIVHSDRLLKSYVDCLLEKGKCTPDGLELKKNMPDAIATDCS
KCSEKQREGSEYMMRFLIDNKPDYWNPLQEKYDPSGAYKQRYLESKKQEVKVEPITKT
>ARM20145.1 GdauCSP9 chemosensory protein 9 [Galeruca daurica]
MNFFCVSILIGFLMVVAVSGAPKTFEENVATLKKVDLKAVLQNTRIMRAYVDCVVHNTHCTPESTALKESWKEG
LDQGCADPCDEEAKKRVQIIAKYVYTEQPEWYKEIIEALDKDKKI
>ARM20143.1 GdauCSP7 chemosensory protein 7 [Galeruca daurica]
MKTFLLIFIAATGSYVFAEKYTTKYDNVDIDTIIKSDRLLLNYVNCLLDRGKCTPDGLELKKVLPDALLTDCSKCSET
QKKGSKKIIRHLIDNKPNWYKELEEKYDKNGTYKIKYEKEIRA
>ARM20139.1 GdauCSP3 chemosensory protein 3 [Galeruca daurica]
MNRWCISVVLMVLVVVAAVHCAPKSFDENLKVLKKIDINQVLNNDRIIRNYVDCVLGKKRCTNEGNALKESWK
DGLDKGCDDCDEEDKRKVKKILKHMYTKHRDLYDELAAHLDKDGKYRDKYQAQIDEILKDPTL
>ARM20138.1 GdauCSP2 chemosensory protein 2 [Galeruca daurica]
MNILRNARMLKRYVECLLDVPDTCTKDGDYLKQVLPEAIKTNCAECDAKQDDTAVRVMAYLMKYHDDWWKK
IDARYQASTSEFLLARKAKIEEYKNTL
>ARM20137.1 GdauCSP1 chemosensory protein 1 [Galeruca daurica]
MKLSLVVCVILAVVVVSAKPEEKYTTKYDNINIDEIINNDRLLRSYVDCLLGTKPCTKDGEELKKVFKEALDSKCSK
CSDAQKEAAKKIGIYLIKNKRPWFDELVEVFDPDHTKLEQYKDELKAEGIEL
>ARM20144.1 GdauCSP8 chemosensory protein 8 [Galeruca daurica]
MKLAIVVCVSLVIVAVAAAPADEKYTSKYDNINLDDIIKSDRLLRNYIDCLLGTKKCTKDAEELKRVLPDALKTKCAK
CTEAQKNGAKKILRHLLKNKREWFNELEAVYDPEHVYVKSYEKELKEEGIEL
>ADX96028.1 HoblCSP1 chemosensory protein CSP1 [Holotrichia oblita]
MRLIFYSFLIYLAIADERYTTKYDNIDIDAVISTERLLQNYIKCLLDLVVCTEEGSELKKNMPDAIQNDCSKCSDKQK
EGSDKLILYLINNKPEYWQLLEEKYDPTGENTKKFIEFKRMMTERTAMYATVSK
>AIX97045.1 MaltCSP5 chemosensory protein 5 [Monochamus alternatus]
MKLVVFLFFVALCGITYGRPDDKYTTKYDNIDLDEILKNDRLLRAYVDCLLGTKKCTNDGEELKKVLPEAIDNECA
KCSEKQKDGAKKVVHYLIDNKSDWWKELEVVYDPTGKYRKKYEEQAKKEGLKI
>AIX97040.1 MaltCSP8 chemosensory protein 8 [Monochamus alternatus]
MLKMIIYFLVAIGLITYATSTVTERTKYTTKYDNIDLDEIIHNERLLKNYVDCLLEKGRCTPDGLELKKNMPDAIETD
CSKCSEKQKEGSEIIIRYLIDNKPEYWNPLQDKYDPTGSYKKRYLDAKKTEVNVEPIVKS
>AIX97041.1 MaltCSP1 chemosensory protein 1 [Monochamus alternatus]
MHFKCQIALLLCIAVMTIVTEAFPQADTERSEVSDEALESTLKDKRYLMRQLKCALGEAPCDPVGRRLKSLAPLVL
QGSCSQCSPKELNQIRKVLSYMQINFPKEWNRVLKQYSR
>AIX97046.1 MaltCSP6 chemosensory protein 6 [Monochamus alternatus]
MVCLLPVLVTTGTLLGLIDGANAQYYASKYDHIDVDAILNNRRIVNYYAACLLTKGPCPPEGIEFKRILPEALRTNC
QRCTEKQKTVTLRAIRRLKKEYPKVWAQLEKQWDPDGSYISKFESTFGAKSTESIPESSVQIVNRYASPEDNKTIE
NALSQIFTTPLPTSTSTSTSTTSTTTATTSTTSTTAKTTTTSKATTVSTKAAPVTKTTTHRVSTINIIKNINTISSSTKPT
KRPRPSIGQSIQATVSIGTNIVGDLVRGLEAIGNRVVETGAEIAGVVLKNIARPL
>AIX97085.1 MaltCSP10 chemosensory protein 10, partial [Monochamus alternatus]
MLYLSSLICLVCISSVIGQKYSNKYDHIDIDRILSSKRVLNNYIKCILDQGPCTPDGREFRDHIPEAITTNCVKCTDA
QVKIIRKTSAHIMNNRPEDWEKIKNKFDPEEKYKDSFMKFIDG

>UMT69261.1 OcomCSP9 chemosensory protein 9 [Ophraella communa]
MGFARLIFLLAVVSYSFAQTYNTRYDNIDIDRILASKRLLDSYIHCLLDDNFKKCSPEGREFKKYIPEAIHTNCGKCS
DSQKRIVKKAAKHIIQNRPQDWDKIRKKYDPERKYQQSFNTFLNSP

>UMT69263.1 OcomCSP11 chemosensory protein 11 [Ophraella communa]
MQTFQPGLILLIVFVSVIVVKAAITEKSSSTERPSISDEALEASLKDKRYLLRQLKCAIGEAACDSVGRRLKSLAPLVL
QGSCSQCTPQEQRQIRKVLGYMQVNFPKEWNKILKQYSG

>APC94216.1 PmacCSP3 chemosensory protein 3 [Pyrrhalta maculicollis]
MVPLICAIVVGLSGLVVPAPVLEDGEQYYTTKYDHVDIEMILNNRRLVNYYAACMLNKGPCPPEGLEFKRILPDA
IQTNCIKCTEKQKATTLRAIKRLMKEYKKIWDQLKAEWDPDDVFVKKFLETYNKPTSTVSNIFSNRFDDEEVSEQ
TQTPLNKPKEPMKSSTAPSTSTTSTTSTTSATLGVFSPPKTTRAPAPNAVNPLANTLGQTIKATVSFGNNIARQVI
KDIETIGNTVISTGVEIGNRVIQTGTQIAGALTGIPRPVNRFNN

>APC94294.1 PaenCSP1 chemosensory protein 1 [Pyrrhalta aenescens]
MKFVLVFVVLGVIAVTARPEDDKYTTKYDNINIDELIQNDRLLRNYVDCLLGTKPCNKDGEELKKVLKDGLRTKC
AKCNDTQKEAAKKLAIHLIKNKKPWFDELTPVYDPDHTIVELYREDLKAEGIEL

>APC94212.1 PmacCSP1 chemosensory protein 1 [Pyrrhalta maculicollis]
MKFVLVFVVLGVIAVTARPEDEKYTTKYDNINIDEIINNDRLLRNYVDCLLGTKSCNKDGEELKKVLKDGLRTKCA
KCNDTQKEAAKKLAIHLIKNKKPWFDELTPVYDPDHTIVELYREDLKAEGIEL

>APC94213.1 PmacCSP8 chemosensory protein 8, partial [Pyrrhalta maculicollis]
KYDNINIDEIIQNDRLMRNYVDCLLGTKPCSKDGEELKKVFKEGVDSKCAKCNDKQKEAAKKIAVHLIKNKRQW
FDELSAVFDPDHSKIEEYREDLKAEGI

>EFA07417.1 TcasCSP3 chemosensory protein 3 [Tribolium castaneum]
MLFTVFLVLTCAHVVFLEEYVIPDNIDIDDILSNERLLKNYVNCLLDKGRCTPEGKKLKSTIPEALSTDCAKCNEKVK
ANVRKVLHHLIDNKPDMWKQLEAKYDPSGEYRSKYKDELEKNGIHV

>EFA07418.1 TcasCSP4 chemosensory protein 4 [Tribolium castaneum]
MFKVLFVVFACVQAYVYAEEYTVPQNIDIDEILKNDRLTKNYLDCILEKGKCTPEGEELKKDIPDALQNECAKCNE
KHKEGVRKVIRHLIKNKPSWWQELQEKYDPKGEYKSRYNHFLEEEGLN

>EFA07419.1 TcasCSP5 chemosensory protein 5 [Tribolium castaneum]
MTAIVFLLALACLKTYVSSQEYLVPQNIDVDEILKNDRLTRNYLDCVLGKGKCTPEGEELKKDIPEALQNGCAKCN
EKHKEGVRKVIHHLIENKPNWWQELESKFDPQGEYKKKYDELLKKEGLAN

>EFA07420.1 TcasCSP2 chemosensory protein 2 [Tribolium castaneum]
MFATSALFAFICIQGLVSAEEYLVPQNIDLDEILKNDRLTRNYIDCILGKGKCTPEGEELKRDIPEALQNECAKCNEK
HKEGVRKVLHHLIKNKPNWWQELEAKFDPKGEYKQKYNKLLEKEGLQA

>EFA07421.1 TcasCSP6 chemosensory protein 6 [Tribolium castaneum]
MFLAIVLVVCACTNVLSEEYTNQYNDELDAALKSERLMKSYFECLLGTGKCTPSGEELKKDIPDALKNECAKCND
KHKEGIRKVIHYLVKQKPEWWEQLQKKFDPQGIYKKRYQNYLDKEGLKA

>EFA07640.1 TcasCSP8 chemosensory protein 8 [Tribolium castaneum]
MIFKIHFLVFGALLTYVSSVEYLILREIDTILKNDQMTRNYLDCVLDKGKCTKEAEKLKKGITETMKNGCVKCEQK
QKEDVHKVFQHLMIHRPNWWHELETKFNPHHEIKLQHLHQSKFNPHEEVKLQHLHQFPHHDFLE
REGFIR

>KYB26756.1 TcasCSP13 chemosensory protein 13 [Tribolium castaneum]
MIPLIAIAGILAVSAAPAEFYESRYDHLDVESILNNRRMVNYYAACLLSKGPCPPQGVDLKRVLPEALQTNCAKCT
EKQRTAAYRSIKRLKKEYPKIWEQLRAVWDPDDVFIRKFETSFESGKPSGVISTNTSPPSPILSNRFGENEEADAAS
NVISSTPLPPTTSTTTRTTLTTKFTTKPSTKPTNKPVVVTKPPQAPPFATVGANLQATVSFGTNLVGGIVRSLGTLG
SRVVESGTKLANMVISAAIRP

>NP_001039283.1 TcasCSP9 chemosensory protein 9 precursor [Tribolium castaneum]
MTAIVFLLALACLKTYVSSQEYLVPQNIDVDEILKNDRLTRNYLDCVLGKGKCTPEGEELKKDIPEALQNGCAKCN
EKHKEGVRKVIHHLIENKPNWWQELESKFDPQGEYKKKYDELLKKEGLAN
