# Supplementary material for: Antennal Transcriptome Evaluation and Analysis for Odorant-Binding Proteins, Chemosensory Proteins, and Suitable Reference Genes in the Leaf Beetle Pest Diorhabda rybakowi Weise (Coleoptera: Chrysomelidae)
Source: Insects. 2024 Apr 7;15(4):251. doi: 10.3390/insects15040251 (PMC11050234; doi:10.3390/insects15040251)
Supplement: Supplementary file 1 [file insects-15-00251-s001.zip › Appendix C.pdf]

Gene sequence of reference genes in *D. rybakowi*

>OR797776 Actin, complete cds[Diorhabda rybakowi]

ATGTGTGACGACGATGTAGCGGCTCTTGTCGTTGACAATGGTTCCGGTATGTGCAAAGCTGGTTTTGCCGGA
GATGACGCTCCACGTGCCGTCTTCCCATCGATCGTCGGTCGCCCAAGACATCAGGGAGTCATGGTTGGTATG
GGACAAAAAGACTCATACGTAGGAGATGAAGCTCAAAGCAAAAGAGGTATCCTTACTTTGAAATACCCAATT
GAACACGGTATCATCACCAACTGGGATGATATGGAAAAGATCTGGCATCACACTTTCTACAATGAACTCCGTG
TCGCTCCAGAAGAACACCCAGTCCTTCTTACTGAAGCTCCACTTAACCCAAAAGCCAACAGAGAAAAGATG
ACACAAATTATGTTTGAAACCTTCAACACCCCAGCTATGTATGTAGCCATCCAAGCCGTACTTTCATTGTACGC
TTCTGGTCGTACCACTGGTATTGTATTGGACTCTGGAGATGGTGTCACCCACACCGTACCAATCTACGAAGGT
TACGCTCTTCCCCATGCTATCCTCCGTTTGGATTTGGCTGGTCGTGACTTGACTGACTACCTCATGAAAATCCT
TACCGAAAGAGGTTACTCATTCACCACTACAGCTGAAAGAGAAATTGTTCGTGACATCAAAGAAAAACTTTG
CTATGTTGCCCTCGACTTCGAACAAGAAATGGCCACCGCTGCTGCTTCTACTTCCCTCGAAAAGAGCTACGA
ATTGCCTGATGGACAAGTCATCACCATTGGTAACGAAAGATTCCGTTGTCCTGAAGCCCTCTTCCAACCTTCC
TTCTTGGGTATGGAATCTTGTGGTATTCACGAAACCGTCTACAACTCCATCATGAAGTGTGACGTAGATATCC
GTAAGGACTTGTACGCCAACACTGTTCTTTCCGGAGGTACCACCATGTACCCAGGTATTGCTGACCGTATGCA
AAAGGAAATCACCGCTCTTGCTCCATCTACCATCAAGATCAAGATCATCGCTCCCCCAGAAAGGAAATACTCC
GTATGGATCGGTGGATCTATCTTGGCTTCCCTCTCCACTTTCCAACAGATGTGGATCTCCAAACAAGAATACG
ACGAATCCGGCCCTGGAATTGTTCACCGCAAGTGCTTCTAA

>Actin

MCDDDVAALVVDNGSGMCKAGFAGDDAPRAVFPSIVGRPRHQGVMVGMGQKDSYVGDEAQSKRGILTLKY
PIEHGIITNWDDMEKIWHHTFYNELRVAPEEHPVLLTEAPLNPKANREKMTQIMFETFNTPAMYVAIQAVLSLY
ASGRTTGIVLDSGDGVTHTVPIYEGYALPHAILRLDLAGRDLTDYLMKILTERGYSFTTTAEREIVRDIKEKLCYVAL
DFEQEMATAAASTSLEKSYELPDGQVITIGNERFRCPEALFQPSFLGMESCGIHETVYNSIMKCDVDIRKDLYANT
VLSGGTTMYPGIADRMQKEITALAPSTIKIKIIAPPERKYSVWIGGSILASLSTFQQMWISKQEYDESGPGIVHRK
CF

>OR797777 GAPDH glyceraldehyde-3-phosphate dehydrogenase, complete cds [Diorhabda rybakowi]

ATGGTGAAAGTAGGAATAAACGGTTTTGGACGTATTGGTCGTTTGGTGCTTCGCGCTGCCCTTTCCAAAGGC
GTAGAAGTAGTAGCCGTTAACGATCCGTTCCTCGATGTCGATTACATGGTCTACTTATTCAAATTCGATTCAAC
CCACGGTCGTTACAAGGGATGCGTCAACAGCGATGGTAAAAATTTGGTAGTTGATGGTAAACCGATTGCTGT
ATACCAAGAAAAAGATCCCGCCCAAATTCCATGGGGTAAACACGGTGCCGACTATGTAGTAGAATCTACCGG
TGTTTTCACCACAGTCGAAAAGGCAAAGAAACATCTGGACGGTGGCGCTAAGAAAGTTATCATCTCAGCTC
CATCTGCTGACGCTCCTATGTACGTTTGCGGCGTCAATCTCGATTCTTACAACCCAGCTGATCCCGTCATTTCG
AACGCTTCCTGTACCACGAACTGCTTGGCTCCATTGGCTAAAGTCATTCATGATAATTTCGAAATTGTCGAAG
GTTTGATGACAACGGTACATGCCACTACTGCCACGCAGAAGACCGTCGACGGTCCGTCCGGTAAATTGTGG
CGAGACGGTCGTGGTGCCGGACAAAACATCATTCCAGCCTCTACTGGAGCTGCCAAGGCAGTCGGCAAAG
TTATCCCAAGTCTTAATGGTAAGTTGACAGGTATGGCGTTCCGCGTACCAGTCCCTAACGTTTCAGTCGTCGA
TTTGACCGTACGTTTGGGCAAATCAGCGACTTACGACGAAATCAAAGCTAAAGTTAAGGAAGCCGCCGAAG
GTCCCCTAAAGGGCATCTTGGGTTACACCGACGAAGAAGTCGTTTCCAACGATTTCATCGGCGATACTCACT
CATCAGTATTCGATGCCAAAGCCGGTATCCAACTCAACGATAAATTCGTTAAACTCATCTCTTGGTACGATAAC
GAATTCGGTTATTCTTCAAGGGTTATCGATTTGATCTTATTCATCTCTAATAAAGCTTAA

>GAPDH

MVKVGINGFGRIGRLVLRAALSKGVEVVAVNDPFLDVDYMVYLFKFDSTHGRYKGCVNSDGKNLVVDGKPIAV
YQEKDPAQIPWGKHGADYVVESTGVFTTVEKAKKHLDGGAKKVIISAPSADAPMYVCGVNLDSYNPADPVISN

ASCTTNCLAPLAKVIHDNFEIVEGLMTTVHATTATQKTVDGPSGKLWRDGRGAGQNIIPASTGAAKAVGKVIPS
LNGKLTGMAFRVPVPNVSVVDLTVRLGKSATYDEIKAKVKEAAEGPLKGILGYTDEEVVSNDFIGDTHSSVFDAK
AGIQLNDKFVKLISWYDNEFGYSSRVIDLILFISNKA

>OR797778 EF1a elongation factor1 alpha, complete cds[Diorhabda rybakowi]

ATGGGGAAAGAAAAGATTCATATTAACATCGTCGTCATTGGTCACGTAGATTCTGGTAAATCTACCACCACTG
GACATTTGATTTACAAATGCGGTGGTATCGACAAACGTACTATCGAAAAATTCGAAAAAGAAGCCCAAGAAA
TGGGAAAAGGTTCCTTCAAGTATGCATGGGTACTCGATAAACTTAAGGCTGAACGTGAACGTGGTATCACTA
TTGATATTGCTTTGTGGAAATTTGAAACATCTAAGTACTATGTAACCATCATTGATGCTCCAGGGCACAGAGAT
TTCATTAAAAACATGATTACTGGAACTTCACAAGCTGATTGTGCAGTACTTATTGTAGCTGCAGGTACTGGTG
AATTCGAAGCTGGTATTTCCAAGAACGGACAAACACGTGAACATGCACTTCTTGCATTCACCCTTGGAGTGA
AACAACTTATTGTTGGTGTCAACAAAATGGACTCTACTGAACCACCATACAGTGAATCACGTTTTGAAGAAAT
CAAAAAAGAAGTTTCCTCTTATATTAAGAAGATTGGTTACAACCCTGCTGCTGTAGCCTTTGTACCAATTTCA
GGATGGCACGGAGATAATATGTTAGAACAATCCGACAAAATGCCATGGTTCAAGGGATGGAATATTGAACGT
AAAGAAGGAAAGGCTGATGGTAAATGTTTGATTGATGCTTTAGATGCCATCCTCCCTCCAAGTCGTCCAACA
GAAAAACCTCTTCGTCTTCCACTCCAGGATGTCTACAAAATTGGAGGTATTGGAACAGTACCCGTTGGTCGT
GTTGAAACTGGTGTTTTGAAACCAGGTATGGTGGTAGTTTTTGCCCCAGCAAACTTGACCACTGAAGTAAAA
TCTGTTGAAATGCACCACGAAGCCCTTCAAGAAGCTGTACCTGGAGACAACGTTGGTTTTAACGTTAAGAA
CGTCTCTGTCAAAGAATTACGTCGTGGTTATGTAGCTGGAGACACCAAGAACAATCCACCAAAAGGAGCATC
AGACTTTACCGCTCAAGTTATTGTACTGAACCATCCCGGTCAAATCTCCAACGGTTACACTCCTGTCCTCGATT
GTCACACTGCCCACATTGCCTGTAAATTCAATGAAATCAAAGAAAAAGTCGACCGTCGTTCTGGAAAGACAA
CTGAAGAAAACCCCAAAGCCATCAAATCTGGTGATGCTGCCATTGTCAACTTAGTTCCAAGCAAACCAATGT
GTGTAGAATCATTCCAAGAATTCCCACCTCTTGGACGTTTCGCTGTTCGTGACATGAGGCAAACAGTCGCCG
TAGGAGTCATCAAGAGTGTAAGCTTCAAAGACCCCGGTGCAGGAAAAGTTACAAAAGCAGCAGAAAAAGC
ACAAAAGAAGAAATAA

>EF1a

MGKEKIHINIVVIGHVDSGKSTTTGHLIYKCGGIDKRTIEKFEKEAQEMGKGSFKYAWVLDKLKAERERGITIDIAL
WKFETSKYYVTIIDAPGHRDFIKNMITGTSQADCAVLIVAAGTGEFEAGISKNGQTREHALLAFTLGVKQLIVGV
NKMDSTEPPYSESRFEEIKKEVSSYIKKIGYNPAAVAFVPISGWHGDNMLEQSDKMPWFKGWNIERKEGKADG
KCLIDALDAILPPSRPTEKPLRLPLQDVYKIGGIGTVPVGRVETGVLKPGMVVVFAPANLTTEVKSVEMHHEALQ
EAVPGDNVGFNVKNVSVKELRRGYVAGDTKNNPPKGASDFTAQVIVLNHPGQISNGYTPVLDCHTAHIACKFN
EIKEKVDRRSGKTTEENPKAIKSGDAAIVNLVPSKPMCVESFQEFPPLGRFAVRDMRQTVAVGVIKSVSFKDPGA
GKVTKAAEKAQKKK

>OR797779 Tub beta1-tubulin, complete cds[Diorhabda rybakowi]

ATGAGGGAAATTGTTCATATCCAAGCCGGTCAATGCGGTAACCAAATTGGAGCCAAATTCTGGGAAATCATC
TCCGATGAACATGGTATCGACCCCACTGGAGCCTACCACGGAGACTCCGACCTCCAACTCGAAAGAATCAAC
GTTTATTACAATGAAGCGTCTGGAGGAAAATACGTACCAAGGGCAATCCTCGTCGATTTGGAACCCGGTACC
ATGGATTCGGTACGATCCGGTCCTTTCGGTCAAATATTCCGACCAGATAATTTCGTATTTGGTCAATCAGGTGC
AGGTAACAACTGGGCAAAAGGACATTACACAGAAGGTGCTGAATTAGTCGATTCAGTATTGGATGTTGTCA
GAAAAGAAGCCGAATCGTGTGATTGTTTACAAGGATTCCAACTTACACATTCTCTTGGAGGTGGTACCGGAT
CAGGTATGGGTACCCTCCTTATTTCCAAAATTCGTGAAGAATATCCCGACAGGATAATGAACACATATTCAGTA
GTCCCCTCACCAAAAGTATCAGACACTGTAGTAGAACCTTACAATGCGACACTTTCCGTTCATCAATTAGTAG
AAAACACAGACGAAACTTATTGTATAGACAATGAAGCTCTCTACGATATTTGCTTCAGGACTTTGAAACTCAC
GACACCAACATACGGTGACTTGAATCATTTAGTATCACTCACAATGTCAGGAGTAACTACCTGTCTTAGATTCC
CTGGTCAATTGAATGCAGATCTTAGGAAATTAGCCGTAAATATGGTACCATTCCCACGTCTCCACTTCTTCATG

CCCGGTTTCGCTCCTCTTACATCCAGAGGTAGCCAACAATACAGGGCTTTGACGGTTCCAGAACTCACTCAA
CAAATGTTTGATGCCAAGAACATGATGGCCGCTTGCGATCCCAGACACGGAAGGTATCTTACCGTAGCCGCC
GTATTCAGAGGTAGGATGTCGATGAAAGAAGTCGACGAACAGATGCTCAACATTCAAAACAAGAACAGCAG
CTACTTCGTCGAATGGATTCCGAACAACGTTAAGACTGCCGTTTGTGACATTCCTCCTAGAGGTCTTAAGATG
TCTGCCACTTTCATCGGTAACTCCACCGCCATTCAAGAATTGTTCAAACGTATCTCAGAACAGTTCACCGCTAT
GTTCAGGAGGAAAGCTTTCTTGCATTGGTACACCGGAGAAGGTATGGATGAAATGGAATTCACTGAAGCTG
AATCTAACATGAACGATTTGGTATCTGAATACCAACAATACCAAGAAGCCACCGCAGACGAAGACGCTGAAT
TCGATGAAGACCAAGAAGCCGAAGTTGATGAAAACTAA
>Tub
MREIVHIQAGQCGNQIGAKFWEIISDEHGIDPTGAYHGDSDLQLERINVYYNEASGGKYVPRAILVDLEPGTMD
SVRSGPFGQIFRPDNFVFGQSGAGNNWAKGHYTEGAELVDSVLDVVRKEAESCDCLQGFQLTHSLGGGTGSG
MGTLLISKIREEYPDRIMNTYSVVPSPKVSDTVVEPYNATLSVHQLVENTDETYCIDNEALYDICFRTLKLTTPTYG
DLNHLVSLTMSGVTTCLRFPGQLNADLRKLAVNMVPFPRLHFFMPGFAPLTSRGSQQYRALTVPELTQQMFDA
KNMMAACDPRHGRYLTVAAVFRGRMSMKEVDEQMLNIQNKNSSYFVEWIPNNVKTAVCDIPPRGLKMSAT
FIGNSTAIQELFKRISEQFTAMFRRKAFLHWYTGEGMDEMEFTEAESNMNDLVSEYQQYQEATADEDAEFDED
QEAEVDEN
>OR797780 RPL13a ribosomal proteinL13a, complete cds[Diorhabda rybakowi]
ATGACGGGTTTTAGTGAAAAATCCATCCTTATCGATGGCAGAGGCCACTTATTGGGACGATTAGCTGCAATC
GTTTCAAAAACTTTATTACAAGGTAACAAAGTGGTAGTAGTACGATGTGAACAATTGAACATCTCTGGAAAC
TTTTACAGAAATAAGTTGAAGTTTATGTCTTTCCTTCGTAAAAGATGTAACGTTAATCCAGCTCGTGGTCCGTT
CCATTTTAGAGCTCCCTCCAGAATCTTCTGGAAGACAGTTAGAGGAATGTTGCCACACAAAACTGAAAGAG
GAAAGCAAGCTCTTCGTAGATTAAAGGCATACGAAGGAATACCTCCTCCATATGATCGTAGGAAACGTGTTG
TTGTACCTGGAGCTCTTAGAGTGATTTGTCTTAAACCTGGACGTAAATTCTGTCACGTTGGCCGTCTGTCACA
TGAAGTCGGATGGAAGTATCAATCAGTCGTAAGGACATTAGAAAACAAACGTAAAGTTAAGGCCGTCTTGA
CTATTCGTAAACGTGACAAACTCAAGAAACTCACTAAAAAAGCTTCAGAAAAGGTTATCAAACAAACGAAA
CCTTTCACAGAACTCATCAATTCATACGGTTATAATTAA
>RPL13a
MTGFSEKSILIDGRGHLLGRLAAIVSKTLLQGNKVVVVRCEQLNISGNFYRNKLKFMSFLRKRCNVNPARGPFHF
RAPSRIFWKTVRGMLPHKTERGKQALRRLKAYEGIPPPYDRRKRVVVPGALRVICLKPGRKFCHVGRLSHEVG
WKYQSVVRTLENKRKVKAVLTIRKRDKLKKLTKKASEKVIKQTKPFTELINSYGYN
>OR797781 RPS18 ribosomal protein S18, complete cds[Diorhabda rybakowi]
ATGTCTTTGGTAATACCAGAGAAGTTCCAACACATTCTTCGTATCCTTGGTACGAATATCGATGGTAAAAGAA
AAGTACAATTCGCCCTTACAGCTATTAAGGGAGTTGGTAGACGTTACGCTAATATTGTCCTTAAAAAAGCCGA
TGTAAACTTGGACAAACGTGCGGGAGAATGTTCCGACGAAGAAGTTGAAAAAATCATAACAATCATGTCTAA
TCCTCGCCAGTACAAAATCCCCGACTGGTTTTTGAACAGACAGAAAGATATTATTGACGGTAAATACAGCCA
GGTAAGTCCTTCTAATTCTTTAAGAAAAAGTTCATCATCTTTAAATTGGGCTGATTCATAA
>RPS18
MSLVIPEKFQHILRILGTNIDGKRKVQFALTAIKGVGRRYANIVLKKADVNLDKRAGECSDEEVEKIITIMSNPRQY
KIPDWFLNRQKDIIDGKYSQVSPSNSLRKSSSSLNWADS
>OR797782 RPL19 ribosomal protein L19, complete cds[Diorhabda rybakowi]
ATGAGTTCGTTAAAACTTCAAAAGAGGCTAGCAGCCTCTGTTATGCGATGTGGAAGAAAAAAAGTTTGGTT
AGATCCCAATGAAATAAACGAAATTGCAAACACCAACTCAAGACAGAACATTCGCAAATTGATTAAGGACG
GTCTTATCATTAAAAAACCGGTAGCAGTACATTCTCGTGCTAGAGTACGTAAGAACACTGAAGCTCGCAGGA
AAGGAAGGCATTGTGGATTTGGTAAAAGGAAAGGTACAGCCAATGCTCGTACTCCTCAAAAGGAATTATGG

ATACAACGTATGAGAGTCTTACGTCGTTTATTGAAAAAGTACCGTGAAGCTAAGAAAATTGACAGACATCTTT
ACCATTCACTTTACATGAAAGCCAAGGGTAACGTATTCAAGAATAAGCGTGTCCTTATGGAATACATTCATAA
AAAGAAGGCAGAGAAAGCACGTGCCAAGATGTTGGCAGACCAGGCTAATGCCAGAAGATTGAAGGTGAA
ACAAGCTAGGGAAAGACGCGAAGAACGTATAGCTACTAAAAAGCAGGAGGTATTGCAAAATTACCAGAGA
GAAGATGAAGCTGCTGCTGCTAAGAAATAA

>RPL19

MSSLKLQKRLAASVMRCGRKKVWLDPNEINEIANTNSRQNIRKLIKDGLIIKKPVAVHSRARVRKNTEARRKGR
HCGFGKRKGTANARTPQKELWIQRMRVLRRLLKKYREAKKIDRHLYHSLYMKAKGNVFKNKRVLMEYIHKKKA
EKARAKMLADQANARRLKVKQARERREERIATKKQEVLQNYQREDEAAAAKK

>OR797783 SYN6 Syntaxin-6, complete cds[Diorhabda rybakowi]

ATGAAAGAAAGAATCAATATGAATCGGAGTAGGGATAAAGATCGTACTGCGAGGCAGCCTTTGCTGGATAAT
AGTCCCGTTAGAGTTACAAATTCACATGCAACAACAAAATATTTTAAATTGGAGAATGATCTAGATAGTCCTC
AACGACAATTTTTGAGTGATACTCTTAACCAACAACAATATTTAACAAGACAACAAGAAGAACATTTGGAGG
CTATTAGTGATTCTTTAGGTTCTTTGAAAACAGTTTCTAGACACATTGGAGTTGAATTGGACGAACAAGCTG
GAATGCTGGATGAATTTGGGACCGAATTAGAAAATACTGATTCCAAATTGGATTCCACAATGAAAAAGGTTG
CCAAAGTACTACGACTTTCTAATGATCGGAGGCAGTGGACCGTTATAATAATTTTAGTTGTAATTCTTGTGATT
GTGATATTTTTATTTTTTATACTGTGA

>SYN6

MKERINMNRSRDKDRTARQPLLDNSPVRVTNSHATTKYFKLENDLDSPQRQFLSDTLNQQQYLTRQQEEHLE
AISDSLGSLKTVSRHIGVELDEQAGMLDEFGTELENTDSKLDSTMKKVAKVLRLSNDRRQWTVIIILVVILVIVIFLF
FIL

>OR797784 GST glutathioneS-transferase1, complete cds[Diorhabda rybakowi]

ATGGGACCGACTTTATATCATTTTCCACCGAGTGGACCCTCTAGAGGAGCCTATCTAGCTGCAAAAGCTGTAG
GAGTTAATGTTGATTTGCAGATCGTAGATTTATTCAAAAAGGAACAGTACAGTGAAGAATTTATCAAGATTAA
CCCTCAACATACTGTGCCTACGTTAGTTGATGGAGACTTTACTATTTGGGATGGTCATGCTATAGCGGGATATT
TAGCAACAGTTTATGCAAAGGATGAAAATTTGTATCCGAAAGATCCACAACGTAAAGCTCTTGTAGATCAAA
GGCTTTATTTTGATTGTGGTACTCTATATCCACGTATTAGGCAAATTTGTTATCCGATTTTATATATGGGAGAAG
ACCAAATCTATGATGAATATAAATTGCCTTTGGAAGAAGCTTTGGGATTCTTAGATGTTTTCTTAGAAGGAAA
TGATTTTGTAACAGGCAAATATCTAACAATTGCCGATTGTTCATTAGTGGCATCAGTTTCAAGCATAGTGGCT
GTAGGTTGGGATATAAATGCATATTCTAATGTTGTTAATTGGATATCAAGATGTGCTTTGGCAATACCAGACTA
TGAGGAGGTTAATCAAAAAGGAGCAAATATGTATGGTAAAGCAGTCAGAAGTAAACTTGCTCCGGGACAAC
TCTAG

>GST

MGPTLYHFPPSGPSRGAYLAAKAVGVNVDLQIVDLFKKEQYSEEFIKINPQHTVPTLVDGDFTIWDGHAIAGYLA
TVYAKDENLYPKDPQRKALVDQRLYFDCGTLYPRIRQICYPILYMGEDQIYDEYKLPLEEALGFLDVFLEGNDFVT
GKYLTIADCSLVASVSSIVAVGWDINAYSNVVNWISRCALAIPDYEEVNQKGANMYGKAVRSKLAPGQL

>OR797785 RPS15 ribosomal protein S15, complete cds[Diorhabda rybakowi]

ATGGCCGACAAAGTCGAAGAGCAAGTTAAAAAGAAGAGAACTTTCAAAAAATTCACCTTCCGTGGTGTAGA
TTTGGACCAGTTATTGGACATGCCAAACGAACAATTAATGGAATTGATGCATTGTCGTGCTAGAAGGCGTTTT
ACTAGAGGCTTAAAACGCAAACCAATGGCTTTGGTTAAAAAATTGCGTAAAGCCAAGAAAGAAGCTCCTCC
ACTAGAAAAGCCAGAAATTGTTAAAACTCATTTACGTAACATGATCATCGTTCCAGAAATGGTTGGTTCTATT
GTAGGTGTTTACAATGGCAAAGCCTTCACACAGGTTGAAATCAAACCTGAAATGATTGGACATTATCTTGGT
GAATTCTCTTTAACATACAAGCCTGTTAAACACGGTAGGCCAGGTATTGGTGCTACCCACAGTTCTAGATTTA
TTCCTCTCAAATAA

>RPS15

MADKVEEQVKKKRTFKKFTFRGVDLDQLLDMPNEQLMELMHCRARRRFTRGLKRKPMALVKKLRKAKKEAP
PLEKPEIVKTHLRNMIIVPEMVGSIVGVYNGKAFTQVEIKPEMIGHYLGEFSLTYKPVKHGRPGIGATHSSRFIPLK

The Ct values for candidate reference gene of *D. rybakowi* under tissue conditions

Sex	Group		*RPS18*	*PRL13a*	*RPL19*	*ACT*	*SYN6*	*TUB*	*GADPH*	*RPS15*	*EF1a*	*GST*
Male	CT1	He	24.36	23.69	24.93	20.66	31.51	25.83	26.80	26.04	25.62	28.58
			24.54	23.63	25.19	21.32	33.31	26.64	25.77	26.58	26.77	27.72
	CT2		21.34	20.37	21.79	23.29	29.87	25.99	25.59	21.96	21.47	21.66
			21.28	19.72	21.44	23.69	31.16	27.02	21.50	22.02	22.95	23.30
	CT3		20.01	19.38	20.62	16.53	22.53	19.73	21.61	22.07	17.75	24.63
			20.01	19.00	19.62	13.39	23.92	19.54	20.87	21.70	18.31	26.03
	CT1	Th	24.73	25.91	26.68	25.54	31.84	28.09	25.99	25.58	26.20	27.80
			25.67	24.18	26.39	22.81	32.63	26.53	26.47	26.03	27.51	28.69
	CT2		21.87	19.91	23.99	27.25	35.18	28.78	24.58	22.82	28.15	23.33
			23.47	20.77	24.05	27.80	34.08	25.28	26.43	22.79	27.85	25.18
	CT3		20.21	17.42	18.78	23.18	26.47	21.34	22.74	21.03	22.82	22.99
			19.89	18.14	18.50	23.36	26.76	22.61	22.91	21.40	23.00	23.08
	CT1	Ab	23.40	23.77	25.16	23.88	33.89	25.02	26.37	25.06	25.93	27.86
			24.26	23.76	24.71	24.07	33.40	26.96	27.57	25.25	26.65	28.35
	CT2		24.94	21.29	24.18	28.07	32.48	29.23	28.61	23.15	26.41	23.66
			22.73	21.39	24.50	27.15	32.25	29.96	28.44	23.20	28.04	24.60
	CT3		22.22	21.35	21.66	23.29	29.88	25.65	27.81	23.03	27.33	23.90
			23.12	21.09	22.53	22.81	30.69	27.46	26.73	23.24	28.11	24.71
	CT1	Le	25.49	25.77	27.02	22.96	33.86	27.65	26.95	27.62	26.49	30.31
			26.43	25.03	27.74	22.73	33.04	28.35	27.29	27.54	27.81	30.58
	CT2		21.91	21.14	22.71	21.61	31.63	28.12	25.99	24.73	23.49	25.20
			22.10	20.86	22.94	22.10	33.40	27.28	24.71	23.79	25.17	24.19
	CT3		20.98	20.03	21.92	18.30	26.98	21.68	22.44	22.49	21.79	25.17
			21.23	19.70	22.26	18.13	27.03	21.81	23.40	23.04	22.36	25.58
	CT1	Wi	23.88	23.03	24.65	26.21	32.74	24.82	26.73	25.20	23.90	27.97
			23.06	22.54	23.71	25.71	32.02	24.75	26.66	25.68	24.12	27.61
	CT2		20.91	18.80	19.40	23.96	28.19	22.60	23.66	20.92	20.48	22.66
			20.82	18.12	19.26	23.67	28.24	23.00	23.52	20.84	21.72	22.97
	CT3		21.74	21.87	23.17	24.08	29.16	23.29	26.33	23.13	21.45	27.12
			22.40	22.39	24.00	23.60	28.54	23.53	27.61	22.93	22.10	28.24
Female	CT1	He	21.87	20.58	20.80	22.77	30.61	25.07	24.47	22.89	24.61	25.85
			22.19	20.56	19.99	21.38	30.94	25.20	24.32	23.16	24.86	25.93
	CT2		23.25	21.26	21.83	25.22	31.03	28.46	26.45	25.11	28.46	23.86
			22.86	21.22	22.23	24.93	31.54	27.38	26.25	24.59	28.05	24.41
	CT3		22.38	21.19	21.47	24.17	31.72	25.30	25.64	23.30	25.47	25.30
			22.02	21.02	21.63	24.04	30.17	25.52	25.65	23.80	25.12	25.71
	CT1	Th	20.53	20.02	20.90	23.39	30.69	26.42	26.09	22.58	26.35	24.76
			20.65	19.86	20.69	23.04	30.52	26.83	25.86	22.85	27.25	23.11
	CT2		21.84	20.03	21.31	23.80	29.95	26.65	25.43	23.28	27.21	21.87
			22.10	20.14	20.58	23.91	29.89	26.47	26.91	21.40	27.40	22.76
	CT3		23.54	22.43	21.58	24.26	31.94	28.52	26.40	23.74	28.24	25.14
			22.72	21.94	21.43	24.34	33.12	28.37	25.52	24.46	28.33	25.66
	CT1	Ab	22.11	21.57	22.36	26.56	31.58	27.01	26.31	23.79	28.04	24.66
			22.75	20.53	22.67	26.32	31.79	27.31	27.28	24.32	28.35	24.94
	CT2		21.46	18.62	20.65	29.07	27.92	25.55	28.45	23.99	25.00	22.13
			21.13	19.15	21.07	28.72	29.44	25.59	28.09	23.53	25.25	22.31
	CT3		21.70	19.86	22.16	26.46	29.88	24.74	28.48	22.15	22.85	24.07
			21.37	20.63	22.23	26.77	30.38	24.47	28.55	22.37	22.85	23.93
	CT1	Le	21.24	20.64	21.86	20.50	31.77	27.46	27.08	22.64	24.00	24.16
			21.59	21.19	21.88	20.47	35.17	27.27	26.59	28.00	23.65	24.36
	CT2		19.66	20.39	21.24	16.96	26.34	21.54	22.22	21.90	21.95	21.99
			19.12	19.83	20.40	16.79	26.83	21.11	22.17	20.61	22.49	21.84
	CT3		21.95	19.26	20.53	15.61	26.18	20.52	23.26	22.09	18.25	24.52
			19.69	19.75	19.89	15.45	26.21	20.15	20.71	21.62	19.11	25.17
	CT1	Wi	21.63	21.01	22.17	27.23	31.21	25.81	26.09	23.10	21.79	25.82
			22.18	21.07	22.39	27.27	31.95	26.07	26.46	23.83	23.42	26.10
	CT2		18.78	18.83	20.97	23.23	27.49	22.05	25.24	20.14	18.61	25.38
			20.67	19.56	21.64	23.49	27.57	21.46	25.16	20.81	19.87	24.84
	CT3		19.33	20.10	19.86	22.81	26.04	20.57	22.07	21.63	16.92	25.62
			20.15	18.26	20.17	22.88	25.38	19.77	26.27	21.50	18.61	25.47

The Ct values for candidate reference gene of *D. rybakowi* under sex conditions												
Sex	Group		*RPS18*	*PRL13a*	*RPL19*	*ACT*	*SYN6*	*TUB*	*GADPH*	*RPS15*	*EF1a*	*GST*
Male	CT1	AD	24.59	24.26	26.18	23.92	34.82	26.52	26.26	26.24	26.15	` 24.36
			24.52	25.29	26.05	24.28	34.02	26.45	26.74	26.03	25.43	28.01
	CT2		21.65	20.62	22.49	23.62	32.22	26.82	25.46	22.68	24.89	23.33
			21.41	20.99	21.65	23.15	31.57	26.78	26.85	22.82	24.00	24.54
	CT3		19.16	18.18	19.18	16.73	24.39	17.58	19.73	19.92	17.36	24.31
			18.62	18.37	18.78	16.75	24.36	19.27	20.82	20.33	17.47	24.69
Female	CT1	AD	22.41	20.39	22.09	23.87	32.29	26.67	26.66	24.41	27.00	24.29
			22.80	20.28	21.78	23.41	32.61	26.27	24.82	21.72	27.08	23.72
	CT2		20.40	17.37	20.18	18.70	26.82	18.96	20.96	20.53	17.90	25.84
			20.78	18.03	20.90	18.48	24.02	19.05	23.63	20.32	15.61	23.35
	CT3		16.43	16.82	18.19	17.80	22.68	17.81	22.84	20.68	17.65	21.64
			17.50	16.72	17.77	18.06	22.13	17.26	23.82	20.93	17.19	22.09
