# Supplementary material for: Antennal Transcriptome Evaluation and Analysis for Odorant-Binding Proteins, Chemosensory Proteins, and Suitable Reference Genes in the Leaf Beetle Pest Diorhabda rybakowi Weise (Coleoptera: Chrysomelidae)
Source: Insects. 2024 Apr 7;15(4):251. doi: 10.3390/insects15040251 (PMC11050234; doi:10.3390/insects15040251)
Supplement: Supplementary file 1 [file insects-15-00251-s001.zip › Figure S4.pdf]

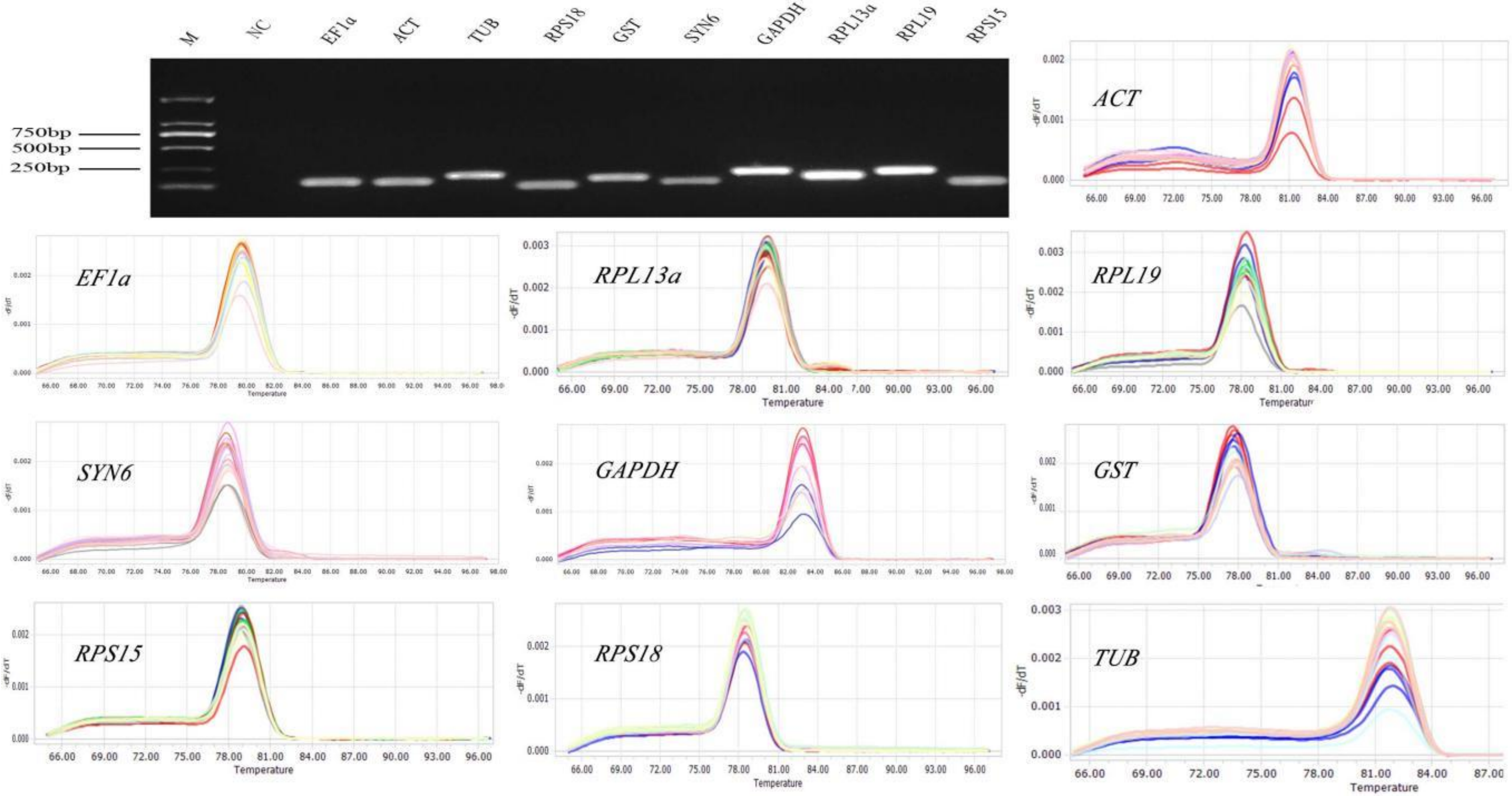

Figure S4. Primer validation and melting curves of ten candidate reference genes. Agarose gel (1.5%) electrophoresis showed amplification of individual PCR products of the expected size for ten genes (lines 1-10: *ACT*, *EF1a*, *RPL13a*, *RPL19*, *SYN6*, *GAPDH*, *GST*, *RPS15*, *RPS18*, and *TUB*). M represents the size position of the 2000 bp DNA marker.
