# Supplementary material for: Antennal Transcriptome Evaluation and Analysis for Odorant-Binding Proteins, Chemosensory Proteins, and Suitable Reference Genes in the Leaf Beetle Pest Diorhabda rybakowi Weise (Coleoptera: Chrysomelidae)
Source: Insects. 2024 Apr 7;15(4):251. doi: 10.3390/insects15040251 (PMC11050234; doi:10.3390/insects15040251)
Supplement: Supplementary file 1 [file insects-15-00251-s001.zip › Table S1.pdf]

Table S1. Assembly summary of antennal transcriptome in *D. rybakowi*

Clusters	Sample name	Raw reads	Clean reads	Error rate (%)	Q20 (%)	Q30 (%)	Unigene		
							Total	N50	Median
Male	ANM_1	53,816,214	51,667,180	0.03	97.60	93.02	51,124	2,148	591
	ANM_2	55,433,876	53,204,984	0.03	97.39	92.53			
	ANM_3	38,744,988	38,134,492	0.02	98.18	94.26			
Female	ANF_1	51,985,680	49,939,080	0.03	97.19	92.10			
	ANF_2	55,560,910	53,360,574	0.03	97.35	92.46			
	ANF_3	40,729,506	40,136,438	0.02	98.26	94.48			
