# Supplementary material for: Antennal Transcriptome Evaluation and Analysis for Odorant-Binding Proteins, Chemosensory Proteins, and Suitable Reference Genes in the Leaf Beetle Pest Diorhabda rybakowi Weise (Coleoptera: Chrysomelidae)
Source: Insects. 2024 Apr 7;15(4):251. doi: 10.3390/insects15040251 (PMC11050234; doi:10.3390/insects15040251)
Supplement: Supplementary file 1 [file insects-15-00251-s001.zip › Table S2.pdf]

Table S2. Primer sequences, product sizes, and PCR efficiencies of ten candidate reference genes in *D. rybakowi*

Gene name	Primer name	Primer sequence (5′→3′)	Amplification Size (bp)	Efficiency (%)	R^2	Linear regression equation
ACT	Forward Primer	CGCCAACACTGTTCTTTCCG	128	105.1	0.99	Y=-3.2057X+23.736
	Reverse Primer	ATTTCCTTTCTGGGGGAGCG				
TUB	Forward Primer	CCGACCTCCAACTCGAAAGAA	180	97.7	0.99	Y=-3.3789X+27.063
	Reverse Primer	TGTTACCTGCACCTGATTGACC				
RPS18	Forward Primer	GACAAACGTGCGGGAGAA	102	99.3	0.97	Y=-3.3378X+24.737
	Reverse Primer	CTGTTCAAAAACCAGTCGGG				
GST	Forward Primer	CAAGCATAGTGGCTGTAGG	153	106.8	0.90	Y=-3.1684X+31.616
	Reverse Primer	CCGGAGCAAGTTTACTTCTGAC				
SYNT6	Forward Primer	GTTGAATTGGACGAACAAGCTG	126	131.8	0.97	Y=-2.7391X+27.258
	Reverse Primer	GCCTCCGATCATTAGAAAGTCG				
GAPDH	Forward Primer	AGTCCCTAACGTTTCAGTCGTC	203	100.1	0.92	Y=-3.3198X+30.669
	Reverse Primer	TGAGTTGGATACCGGCTTTG				
EF1a	Forward Primer	GACAAAATGCCATGGTTCAAG	136	100.5	0.99	Y=-3.3104X+27.173
	Reverse Primer	GGAGTGGAAGACGAAGAGGTTT				
RPL13a	Forward Primer	CCGTTCCATTTTAGAGCTCCCT	158	108.6	0.98	Y=-3.1321X+22.458
	Reverse Primer	CCAGGTACAACAACACGTTTCC				
RPL19	Forward Primer	GCCAATGCTCGTACTCCTCAA	200	108.1	0.97	Y=-3.1416X+23.163
	Reverse Primer	CGTGCTTTCTCTGCCTTCTTT				
RPL15	Forward Primer	GGAATTGATGCATTGTCGTG	107	102.5	0.92	Y=-3.2634X+24.228
	Reverse Primer	GTGGAGGAGCTTCTTTCTTGGT				
