# Supplementary material for: Antennal Transcriptome Evaluation and Analysis for Odorant-Binding Proteins, Chemosensory Proteins, and Suitable Reference Genes in the Leaf Beetle Pest Diorhabda rybakowi Weise (Coleoptera: Chrysomelidae)
Source: Insects. 2024 Apr 7;15(4):251. doi: 10.3390/insects15040251 (PMC11050234; doi:10.3390/insects15040251)
Supplement: Supplementary file 1 [file insects-15-00251-s001.zip › Table S3.pdf]

Table S3. Primer used in RT-qPCR of *DrybOBPs* and *DrybCSPs*

Gene name	Primer name	Primer sequence (5′→3′)	Amplification Size (bp)
**Odorant binding proteins (OBPs)**			
DrybOBP1	Forward Primer	ATGGTGAATACGAAGGACCG	110
	Reverse Primer	CAACCCAATTTTCTTGTTGATG	
DrybOBP2	Forward Primer	GTGAATTCGAAGGGCCAAATA	165
	Reverse Primer	GCTTAAATTGCAGGGATTTTCTG	
DrybOBP3	Forward Primer	CACGCTCTATGTATGAACGTAGGA	182
	Reverse Primer	TCTGACAAACCAACAGCAGC	
DrybOBP4	Forward Primer	TCCAGAACACGAACGCATAA	156
	Reverse Primer	CCTGCTTTGACTGCCATACA	
DrybOBP5	Forward Primer	GCCAATCTGATCCAGCATCGTA	188
	Reverse Primer	ACTATTTCCTGCACTCTCGAGG	
DrybOBP6	Forward Primer	GCAAAACGAACAGGGTGACTAC	174
	Reverse Primer	GAATCTATCTTCTTGGCACAATCA	
DrybOBP7	Forward Primer	GGCAAAGGGCTGAATGAAAAATT	158
	Reverse Primer	TACGTCTTATCTACGAACCTGGG	
DrybOBP8	Forward Primer	CTGGCTATAAGTAACGCTAAGCC	192
	Reverse Primer	ATCTGCCCCGATGTATCC	
DrybOBP9	Forward Primer	GGTTTGATGTATCCCTCTGGAGA	126
	Reverse Primer	GGAAATAGAAGAAGGTCGAGCAC	
DrybOBP10	Forward Primer	TGATGTGATGGAAACTATGAAGG	106
	Reverse Primer	GCAATTATCGAGGGCTGCTA	
DrybOBP11	Forward Primer	TGACCGATATCAAAAATGCTACGA	170
	Reverse Primer	TGCAGGTGGACCTTCTGTTAA	
**Chemosensory proteins (CSPs)**			
DrybCSP1	Forward Primer	TCAGGGAAGCGTTGGAAAC	141
	Reverse Primer	GGAGTGGTCTGGATCGAAAACT	
DrybCSP2	Forward Primer	TTCTACCGGACGCATTGAAAAC	149
	Reverse Primer	CAGATAAACGTGGTCGGAATCG	
DrybCSP3	Forward Primer	ATTGATTGTGGTAGCAGCAGTG	189
	Reverse Primer	CCGTCTTTCCAGCTTTCTTTCA	
DrybCSP4	Forward Primer	GGCGTTGGTATCTTGTAGTTTAGC	151
	Reverse Primer	TCCCGTCCTTCTGGTGAAC	
DrybCSP5	Forward Primer	CTACGACTCTAAGACCTTCCATCTC	159
	Reverse Primer	TTCCTAATTTGTTGTTGCTCCTG	
DrybCSP6	Forward Primer	CCAGATGCCATACGAACTAATTG	226
	Reverse Primer	CGTCAGATTCTTCACCACCAT	
