# Supplementary figures and images for: Antennal Transcriptome Evaluation and Analysis for Odorant-Binding Proteins, Chemosensory Proteins, and Suitable Reference Genes in the Leaf Beetle Pest Diorhabda rybakowi Weise (Coleoptera: Chrysomelidae)
Source: Insects. 2024 Apr 7;15(4):251. doi: 10.3390/insects15040251 (PMC11050234; doi:10.3390/insects15040251)

Gene Function Classification (GO)

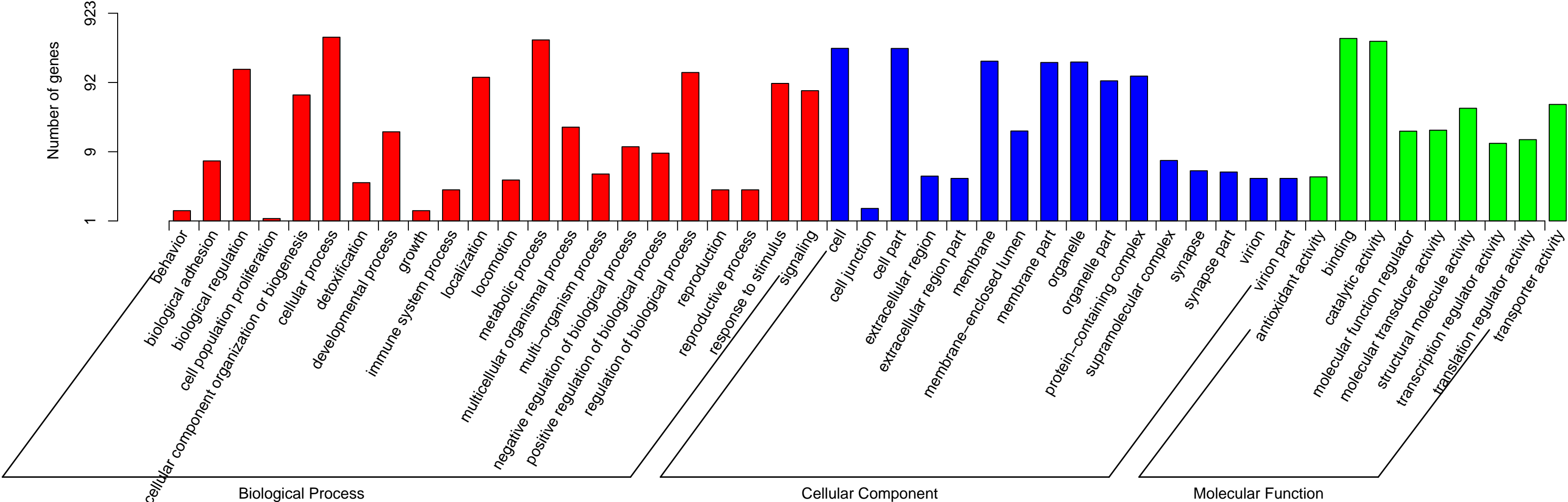

Supplement: Supplementary file 1 [file insects-15-00251-s001.zip › Supplementary Materials Figure/Figure S1. Gene ontology (GO) assignment of D.rybakowi unigenes.pdf]

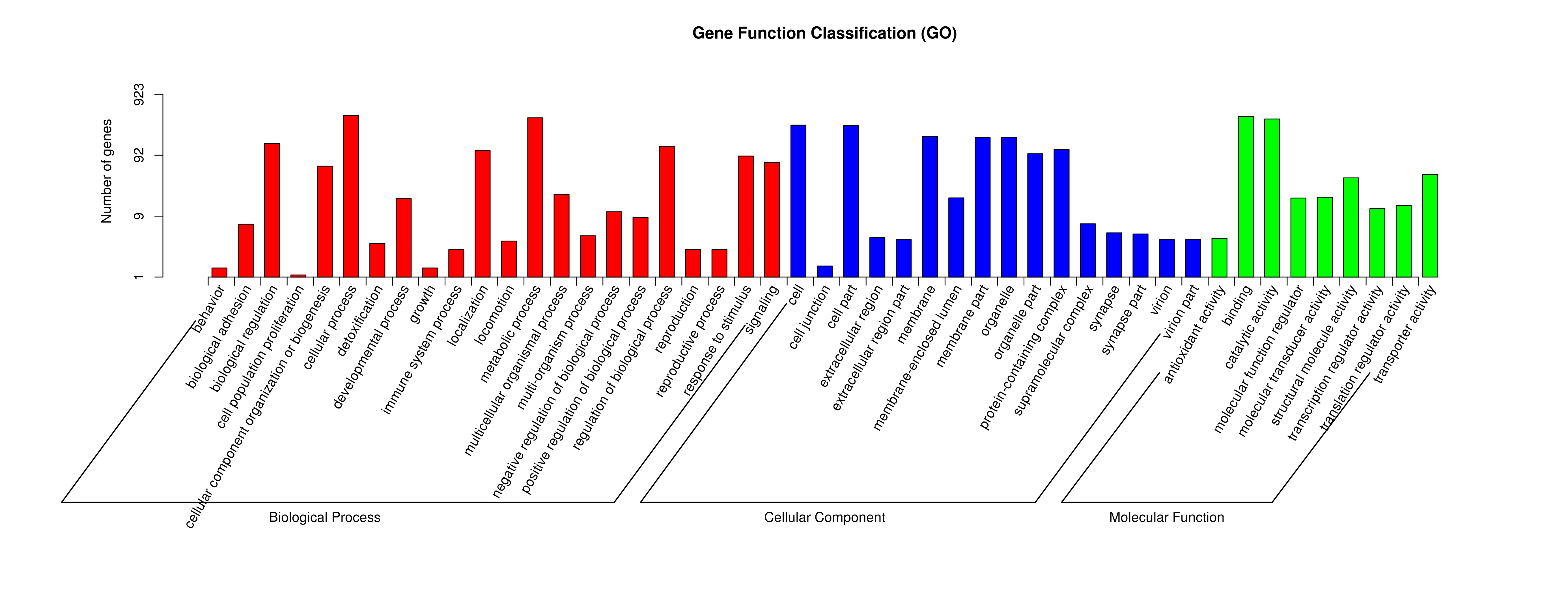

Supplement: Supplementary file 1 [file insects-15-00251-s001.zip › Supplementary Materials Figure/Figure S1. Gene ontology (GO) assignment of D.rybakowi unigenes.png]

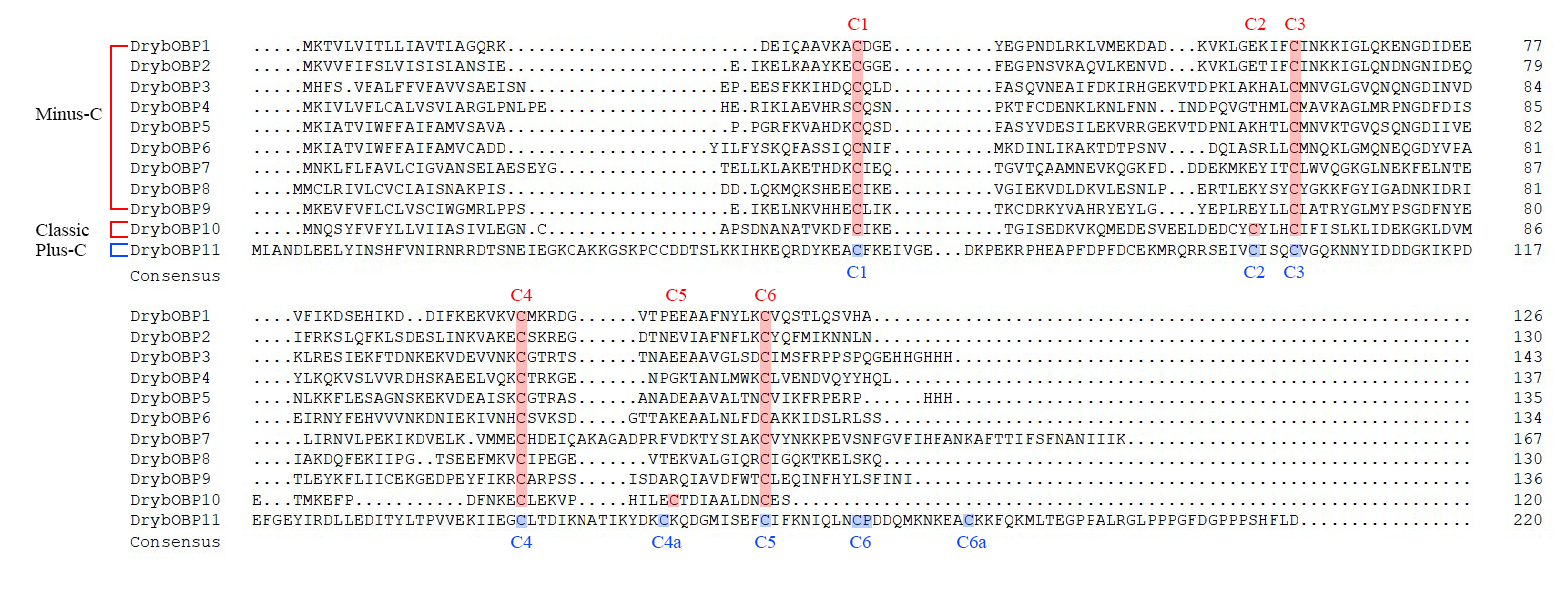

Supplement: Supplementary file 1 [file insects-15-00251-s001.zip › Supplementary Materials Figure/Figure S2. Sequences alignment of OBPs in D. rybakowi.tif]

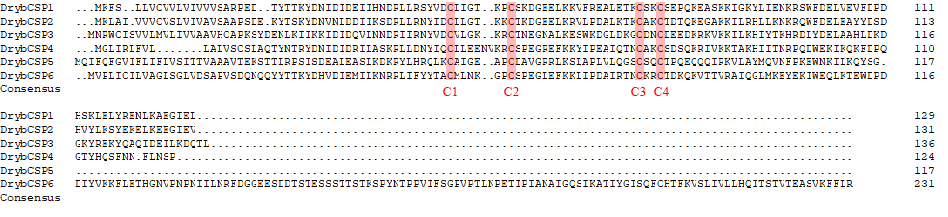

Supplement: Supplementary file 1 [file insects-15-00251-s001.zip › Supplementary Materials Figure/Figure S3. Sequences alignment of CSPs in D. rybakowi.tif]

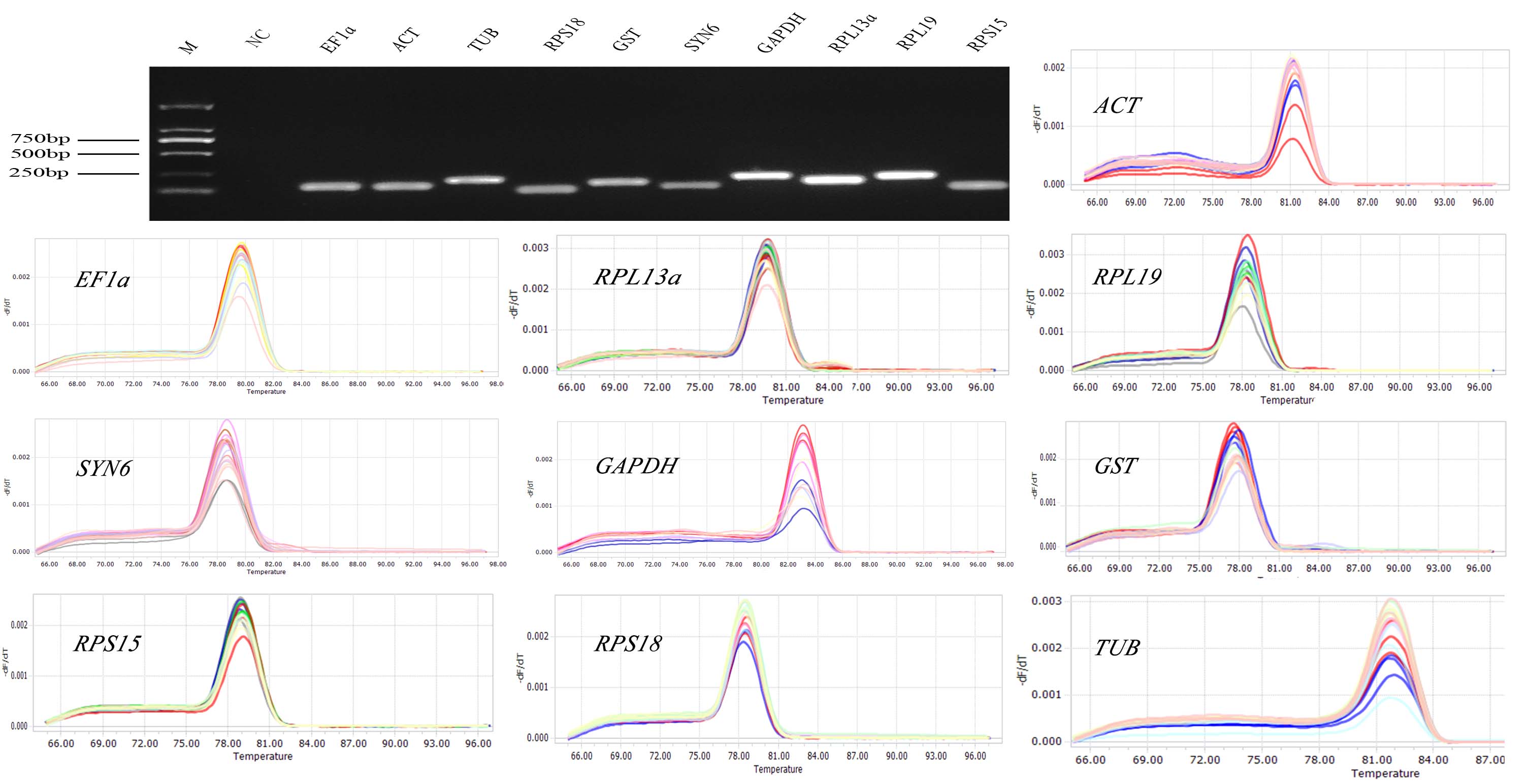

Supplement: Supplementary file 1 [file insects-15-00251-s001.zip › Supplementary Materials Figure/Figure S4. Primer validation and melting curves of ten candidate reference genes.jpg]
